# Unravelling the role of inflammatory markers in coronary artery disease risk via association, mediation and prediction analyses

**DOI:** 10.7189/jogh.16.04060

**Published:** 2026-02-13

**Authors:** Hao Zhang, Yuxin Liu, Yu Yan, Jike Qi, Hua Lin, Yuchen Jiang, Xinyi Wang, Hongyan Cao, Zhou Jiang, Shuo Zhang, Ting Wang, Yue Xu, Weiyi Song, Ke Wang, Chu Zheng, Ping Zeng

**Affiliations:** 1 Department of Biostatistics, School of Public Health, Xuzhou Medical University, Xuzhou, Jiangsu, China; 2 Department of Health Statistics, Shanxi Provincial Key Laboratory of Major Diseases Risk Assessment, School of Public Health, MOE Key Laboratory of Coal Environmental Pathogenicity and Prevention, Shanxi Medical University, Taiyuan, Shanxi, China; 3 Department of Epidemiology and Biostatistics, School of Public Health, Tongji Medical College, Huazhong University of Science and Technology, Wuhan, Hubei, China; 4 Jiangsu Engineering Research Center of Biological Data Mining and Healthcare Transformation, Xuzhou Medical University, Xuzhou, Jiangsu, China

## Abstract

**Background:**

Systemic inflammation plays a critical role in coronary artery disease (CAD), yet comprehensive profiling of inflammatory markers and their integration into predictive models remain incompletely characterised. We here sought to identify key CAD-related inflammation markers and construct an integrated inflammatory risk score (IRS) to enhance conventional cardiovascular risk prediction.

**Methods:**

Associations between 18 complete blood count based inflammatory markers and incident CAD were assessed among 475 134 UK Biobank participants free of CAD at baseline. Weighted quantile sum (WQS) regression evaluated the relative directional contributions of individual markers, and mediation analysis further examined the role of inflammation in linking accelerated aging and unhealthy lifestyle factors to CAD. Predictive performance was assessed by comparing IRS-augmented models in terms of AUC (area under the receiver operating characteristic curve), net reclassification improvement (NRI), and decision curve analysis.

**Results:**

All analysed inflammatory markers showed statistically significant associations with CAD risk, although effect sizes varied. Monocyte-to-HDL-C ratio (MHR), neutrophil-to-HDL-C ratio (NHR), and systemic inflammation response index (SIRI) exhibited the strongest positive associations (21–41% increased risk per SD), while platelet-to-lymphocyte ratio (PLR) and platelet-to-leukocyte ratio (PWR) demonstrated modest inverse associations (5–13% decreased risk). Several markers (*e.g*. MHR, PLR, PWR) displayed discernible group-level differences over a decade before CAD onset. WQS regression highlighted heterogeneous, direction-specific contributions of these markers to CAD risk. Mediation analysis suggested that a portion of the observed associations may operate through accelerated aging (mediation proportion = 7–43%) or via inflammation in the link between unhealthy lifestyle and CAD. The integrated IRS modestly improved CAD risk prediction, particularly within the short-term window of 0 − 5 years (absolute increase in AUC (ΔAUC) = 2.7%, NRI = 1.6%; net benefit = 5%).

**Conclusions:**

Inflammatory markers captured by routine test were consistently associated with future CAD, suggesting that part of CAD risk is reflected in low-cost hematologic parameters. An integrated IRS showed modest but statistically significant improvement in risk discrimination, but external validation and clinical impact studies are needed before implementation.

Coronary artery disease (CAD) remains a leading global cause of mortality, accounting for over 50% of cardiovascular disease-related deaths and imposing significant burdens on public health and socioeconomic systems [1]. Early detection and management of CAD risk factors can substantially alleviate this public health challenge [2], particularly through the regulation of early inflammatory responses [3]. While traditional CAD biomarkers include acute-phase proteins, cytokines, and cell adhesion molecules [4], recent research has shifted focus to complete blood count (CBC) parameters due to their cost-effectiveness and clinical accessibility. Epidemiological evidence has confirmed that circulating white blood cells and their subtypes (*e.g*. monocytes, lymphocytes) are independently associated with CAD risk [5–7], possibly contributing to chronic low-grade inflammation by promoting plaque formation and rupture, endothelial dysfunction, and thus influencing the onset and progression of CAD [8–10].

Emerging studies suggest inflammatory markers combining their derived indices may offer a more comprehensive and accurate CAD risk assessment [11–15], with some proposing that non-invasive and easily accessible indicators, including inflammatory markers, have the potential to supplement or replace invasive angiography for early screening of high-risk individuals prone to CAD [15,16]. Despite growing interest, most studies assessed only one or a few markers in isolation and were limited by small sample sizes and heterogeneous populations, lacking cross-marker comparisons and the assessment of redundancy *vs*. complementarity. Additionally, the incremental value of inflammatory markers beyond established cardiovascular risk models such as Framingham risk score (FRS) [17] and SCORE2 [18] remains unclear.

To address these gaps, we leveraged the UK Biobank [19], a large prospective cohort, to:

(i) perform head-to-head comparisons of up to 18 routine CBC based inflammatory markers and derived ratios with incident CAD within a unified analytic framework;

(ii) characterise group level temporal patterns up to ~ 10 years before diagnosis;

(iii) explore whether accelerated aging [20] and unhealthy lifestyle [21] may partly mediate these associations; and

(iv) evaluate the time-window-specific predictive ability of an integrated inflammatory risk score (IRS), emphasising its incremental value over FRS/SCORE2 (**Figure 1**).

**Figure 1:**
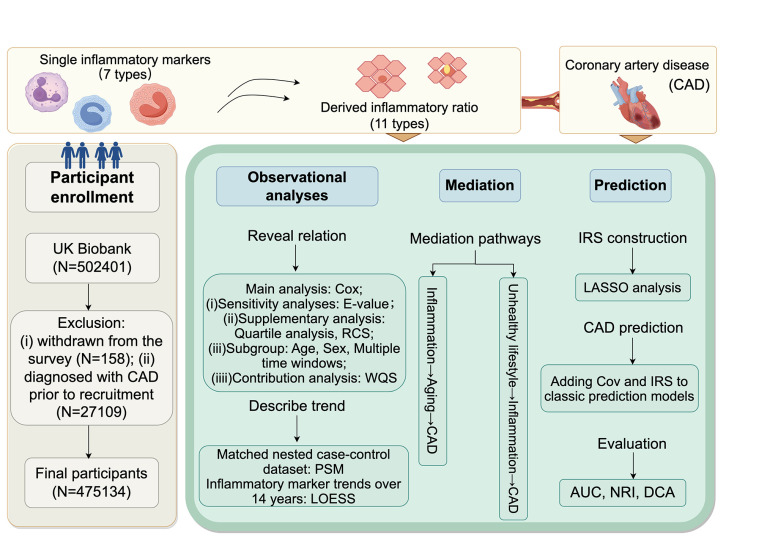
Overview of study design and data analyses. AUC – area under the receiver operating characteristic curve, Cov – covariates, DCA – decision curve analysis, RCS – restricted cubic splines, WQS – weighted quantile sum, PSM – propensity score matching, LOESS – locally estimated scatterplot smoothing, IRS – inflammatory risk score, NRI – net reclassification index.

Our aim is to provide a comparative map of marker performance and a transparent assessment of incremental prediction; translation to practice will require external validation and model recalibration.

## METHODS

### Adherence to JoGH’s Guidelines for Reporting Analyses of Big Data Repositories Open to Public (GRABDROP)

This study is based on secondary analysis of the UK Biobank resource (application number: 88159). We confirm that our work adheres to the Journal of Global Health’s Guidelines for Reporting Analyses of Big Data Repositories Open to Public (GRABDROP) (Table S1 in the **Online Supplementary Document**) [22].

### Definition of CAD and inflammatory markers

CAD cases were identified by first occurrence records (category 1712), which were obtained through the primary care data (category 3000), hospital inpatient data (category 2000), death register records (fields 40001 and 40002) and self-reported medical condition codes (field 20002). Individuals with pre-recruitment CAD were excluded. Follow-up for each participant was tracked from baseline to the earliest of CAD occurrence, death, loss to follow-up, or the censoring date (19 July 2022).

Inflammatory exposures included:

(i) single CBC measures including leukocyte, neutrophil, lymphocyte, monocyte, eosinophil, basophil, and platelet count;

(ii) derived ratios that we grouped a priori into three biological families for interpretation: (A) Leukocyte-centric ratios: neutrophil-to-lymphocyte ratio (NLR), derived neutrophil-to-lymphocyte ratio (dNLR), monocyte-to-lymphocyte ratio (MLR), neutrophil-to-monocyte-lymphocyte ratio (NMLR), systemic inflammation response index (SIRI), and systemic immune-inflammation index (SII); (B) Platelet-anchored ratios: platelet-to-lymphocyte ratio (PLR) and platelet-to-leukocyte ratio (PWR); (C) HDL-anchored ratios: monocyte-to-HDL-C ratio (MHR), neutrophil-to-HDL-C ratio (NHR), and platelet-to-HDL-C ratio (PHR) (Table S2 in the **Online Supplementary Document**).

To minimise the influence of extreme values, inflammatory marker values falling below the 1st percentile or above the 99th percentile of the full-sample distribution were excluded prior to standardisation (Table S3 in the **Online Supplementary Document**). All remaining values were then Z-score standardised.

### Definition of covariates

To account for potential confounding factors, we included a set of commonly recognised covariates that have been previously reported to be associated with both inflammatory markers and CAD [23–25]. These covariates include age, sex, body mass index (BMI), ethnicity, Thomson Deprivation Index (TDI), educational qualification, physical activity, diet score, smoking history, and drinking history (Table S2 in the **Online Supplementary Document**). We also provided the missingness pattern of covariates and imputation strategy (Table S4 in the **Online Supplementary Document****)**.

### Statistical methods and data analyses

#### Link of baseline changes in inflammatory markers with incident CAD risk

Cox proportional hazards (PH) model evaluated the association of baseline inflammatory markers with incident CAD risk. The PH assumption was tested for each marker using Schoenfeld residuals, with *P*-values <0.05 considered evidence of non-proportionality. The association analysis was adjusted for these aforementioned covariates, and the risk was expressed as hazard ratio (HR) with its 95% confidence interval (CI). To examine the robustness of our results, we calculated E-value to evaluate the impact of unobserved confounders [26]. In addition, we restricted CAD cases to those identified solely through ICD-coded hospital or death records, excluding self-reported diagnoses. Second, we applied sex-specific thresholds (1st and 99th percentiles) to define and exclude outliers separately for males and females. Third, we repeated the analyses using complete cases without performing imputation. Lastly, we examined the robustness of the models after adjusting for baseline use of medications (antiplatelet agents, statins, and anticoagulants), excluding participants with major comorbidities (hematologic malignancies, chronic liver diseases, and chronic immune-related disorders), and removing CAD events that occurred within the first two years of follow-up. Quartile-based analyses were performed to assess dose-response trends by comparing the first quartile (Q1) to other three quartiles (Q2−Q4). Restricted cubic splines (RCS) were applied to explore potential nonlinear relationships.

To assess model's discrepancy across subgroups, we performed three stratified analyses:

(i) age-based analysis using a 55-year cutoff to differentiate early-onset from late-onset CAD due to possible distinct mechanisms [27];

(ii) sex-based analysis to account for differences in inflammatory responses and CAD risk between males and females [28];

(iii) time-to-CAD onset analysis by categorising participants into three groups based on disease progression time (0 − 5 years, 5 − 10 years, and >10 years) to evaluate the impact of inflammatory markers on both short-term and long-term CAD risk.

To assess the effect of inflammatory markers as a mixture and determine their relative contributions to CAD risk, we conducted the bidirectional WQS regression, initially designed for assessing multiple chemical exposures in environmental epidemiology [29]. We here adapted WQS to analyse the mixture influence of inflammatory markers and generated their weights to illustrate the individual contributions of each marker.

#### Analyses of inflammatory markers trends prior to CAD diagnosis

To characterise the longitudinal dynamics of systemic inflammation preceding the onset of CAD, we conducted a nested case-control analysis leveraging up to 14 years of follow-up data. Using locally estimated scatterplot smoothing (LOESS) regression [30], we generated the temporal trajectories of inflammatory marker levels across annual time points prior to CAD diagnosis. To reduce potential confounding and ensure comparability, we implemented 1:1 propensity score matching (PSM) to match CAD cases with controls on a comprehensive set of baseline covariates. This matching approach minimised baseline differences, enabling clearer attribution of inflammatory marker trajectories to CAD development itself rather than to pre-existing individual differences [31].

#### Mediation analyses for aging and unhealthy lifestyle

Given that inflammation may play a key role in the aging process [20] and further influence the risk of CAD, we calculated KDM-age [32] as a quantitative measure of aging and included it as a mediator to assess whether inflammatory markers contribute to CAD risk by accelerating the aging process (**Online Supplementary Document**). In addition, as an unhealthy lifestyle is a well-known risk factor for CAD [33], we examined it as the exposure and inflammatory marker as the mediator to understand its effect on CAD risk mediated through inflammation. Following a prior study [34], an unhealthy lifestyle was determined by evaluating seven factors: diet, alcohol use, smoking, physical activity, sedentary behaviour, sleep, and social connection. Each healthy habit earned 1 point, with scores from 0 to 7; a score of 2 or less indicated an unhealthy lifestyle. The proportion of mediation effect was reported, representing the proportion of the total effect mediated through the inflammatory marker.

#### Prediction models for CAD onset

To construct a prediction model of CAD including inflammatory markers, LASSO was first used to select key markers [35]. Subsequently, selected markers were employed to generate a weighted IRS: IRS = β_1_ × X_1_ + β_2_ × X_2_⋯ + β_P_ × X_P_, where X_1_, X_2_,⋯, X_p_ represent the selected markers, and β_1,_ β_2,_⋯, β_P_, denote their effects. To prevent overfitting, all participants were split into three groups: 40% for determine the effect of each selected marker when constructing IRS, 30% for estimating the impact of IRS, and 30% for assessing the IRS's predictive role in CAD events.

We calculated FRS [17], a classic clinical model for predicting the 10-year CAD risk, as well as SCORE2 [18], an updated CAD risk prediction model, both of which served as reference models. Multiple machine learning models, such as logistic, LightGBM and XGboost, were initially assessed for CAD risk prediction. However, due to minimal improvement in predictive performance and the need for interpretability and applicability with clinical use, logistic regression was chosen as the final model, based on which three models were constructed:

(i) model with only FRS or SCORE2;

(ii) model with FRS or SCORE2 as well as other available covariates, which excluded age, sex, BMI, and smoking status as they were already included in FRS or SCORE2;

(iii) model with FRS or SCORE2, covariates and IRS. To assess the predictive performance of these models across different time windows of CAD onset, risk predictions were performed for 0 − 5 years, 5 − 10 years, and >10 years. We compared these models using the area under the receiver operating characteristic curve (AUC) using the DeLong method. To assess reclassification improvement, the net reclassification index (NRI) was calculated after adding covariates and IRS. Clinical utility was evaluated using decision curve analysis (DCA) across threshold probabilities of 0 ~ 30%.

#### Statistical analysis software

Data processing and statistical analyses were conducted using the *R* software version 4.4.1 (R Foundation for Statistical Computing, Vienna, Austria). Missing values were imputed with the mice package. WQS weights were calculated using the gWQS package. Mediation analysis was conducted with the CMAverse package. LASSO analysis utilised the glmnet package. Model performance was evaluated via the pROC package, DCA via the rmda package, and NRI via the nricens package. A Bonferroni correction set a significance threshold at *P* = 2.80 × 10^−3^ ( = 0.05/18) for multiple comparisons. This study adheres to the STROBE reporting guidelines and provide a completed checklist (Table S5 in the **Online Supplementary Document**).

## RESULTS

### Population characteristics

The study included 47 134 UK Biobank participants with inflammatory marker data, the mean age was 56.2 ± 8.1 years old, 44.3% were male, and 94.5% were white. Over a median follow-up of 13 years (interquartile range = 12.6–14.1 years), 39 963 (8.4%) developed CAD, with affected individuals being older, having higher BMI, and often from lower socioeconomic backgrounds with lower education, higher rate of smoking and alcohol use (**Table 1**; Table S6 in the **Online Supplementary Document**). Notably, complex links existed across inflammatory markers; for example, leukocyte counts showed relatively strong correlations with others (Pearson correlation coefficient (*r*) = 0.24–0.90), whereas platelet counts exhibited weak correlations (*r* = 0.01–0.24) (Figure S1 in the **Online Supplementary Document**).

**Table 1:** Baseline characteristics of the participants analysed in our study

Characteristics	All (n = 475 134)	Non-cases (n = 435 171)	CAD cases (n = 39 963)
**Continuous variables, mean (SD)**			
Leukocyte (10^9^ cells/L)	6.9 (2.1)	6.8 (2.1)	7.2 (2.3)
Platelet (10^9^ cells/L)	253.7 (59.8)	254.1 (59.5)	250.2 (62.4)
Lymphocyte (10^9^ cells/L)	2.0 (1.2)	2.0 (1.1)	2.0 (1.4)
Monocyte (10^9^ cells/L)	0.5 (0.3)	0.5 (0.3)	0.5 (0.2)
Neutrophils (10^9^ cells/L)	4.2 (1.4)	4.2 (1.4)	4.5 (1.5)
Eosinophils (10^9^ cells/L)	0.2 (0.1)	0.2 (0.1)	0.2 (0.1)
Basophils (10^9^ cells/L)	0.03 (0.1)	0.03 (0.1)	0.03 (0.1)
NLR	2.3 (1.2)	2.3 (1.2)	2.5 (1.3)
dNLR	0.9 (0.1)	0.9 (0.1)	0.9 (0.1)
SIRI	1.1 (1.1)	1.1 (1.1)	1.3 (0.9)
MLR	0.3 (0.3)	0.3 (0.3)	0.3 (0.2)
NMLR	2.6 (1.3)	2.6 (1.3)	2.8 (1.4)
SII	598.2 (360.6)	595.6 (355.6)	625.9 (410.1)
PLR	142.5 (68.5)	142.8 (69.1)	138.8 (60.7)
PWR	39 (14.0)	39.2 (14.2)	36.3 (11.1)
NHR	3.1 (1.5)	3.1 (1.4)	3.6 (1.6)
MHR	0.4 (0.3)	0.3 (0.4)	0.4 (0.2)
PHR	185 (64.5)	183.8 (63.8)	198.3 (69.9)
Age	56.2 (8.1)	55.9 (8.1)	60.0 (7.0)
BMI	27.3 (4.8)	27.2 (4.7)	28.6 (5)
TDI	−1.3 (3.1)	−1.4 (3.1)	−1.0 (3.2)
Follow-up years, in years	13.0 (2.2)	13.5 (0.9)	7.5 (3.9)
**Categorical variables, n (%)**			
Sex			
*Female*	264 751 (55.7)	249 563 (57.3)	15 188 (38)
*Male*	210 383 (44.3)	185 608 (42.7)	24 775 (62)
Ethnicity			
*White*	449 026 (94.5)	411 278 (94.5)	37 748 (94.5)
*Other*	26 108 (5.5)	23 893 (5.5)	2215 (5.5)
Qualifications			
*No college degree*	292 020 (61.5)	265 101 (60.9)	26 919 (67.4)
*College degree*	183 114 (38.5)	170 070 (39.1)	13 044 (32.6)
Physical activity			
*Low*	89 278 (18.8)	80 869 (18.6)	8409 (21)
*Moderate*	193 671 (40.8)	177 892 (40.9)	15 779 (39.5)
*High*	192 185 (40.4)	176 410 (40.5)	15 775 (39.5)
Diet score			
*0*	8707 (1.8)	7755 (1.8)	952 (2.4)
*1*	41 366 (8.7)	37 269 (8.6)	4097 (10.3)
*2*	93 008 (19.6)	84 431 (19.4)	8577 (21.5)
*3*	130 664 (27.5)	119 486 (27.5)	11 178 (28)
*4*	128 852 (27.1)	118 786 (27.3)	10 066 (25.2)
*5*	72 537 (15.3)	67 444 (15.5)	5093 (12.7)
Smoking history			
*No*	192 667 (40.6)	179 030 (41.1)	13 637 (34.1)
*Yes*	282 467 (59.4)	256 141 (58.9)	26 326 (65.9)
Drinking history			
*No*	457 416 (96.3)	419 576 (96.4)	37 840 (94.7)
*Yes*	17 718 (3.7)	15 595 (3.6)	2123 (5.3)

dNLR – derived neutrophil-to-lymphocyte ratio, MHR – monocyte-to-HDL-C ratio, MLR – monocyte-to-lymphocyte ratio, NLR – neutrophil-to-lymphocyte ratio, NMLR – neutrophil-to-monocyte-lymphocyte ratio, NHR – neutrophil-to-HDL-C ratio, PHR – platelet-to-HDL-C ratio, PLR – platelet-to-lymphocyte ratio, PWR – platelet-to- leukocyte ratio, SIRI – systemic inflammation response index, SII – systemic immune-inflammation index

### Relation between inflammatory markers and CAD

#### Cox analyses revealed significant associations between inflammatory markers and CAD

**Figure 2:**
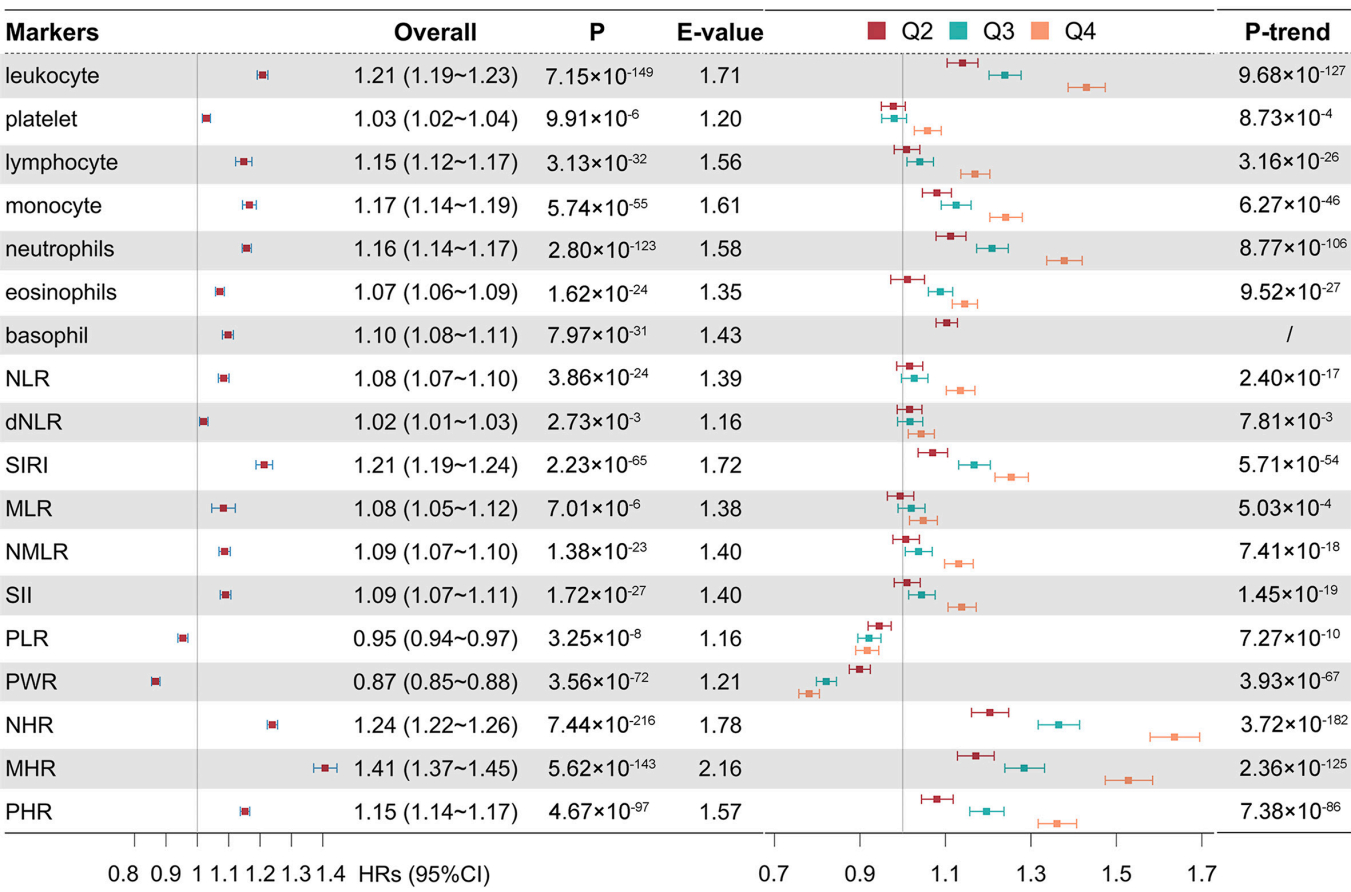
Hazard ratios (HRs) and 95% confidence intervals from Cox proportional hazards models for the associations between inflammatory markers and incident coronary artery disease. ‘Overall’ shows HR per standard deviation increase. E-value indicate the minimum unmeasured confounding strength (risk-ratio scale) required to fully explain away the observed association. Quartile analyses compare Q2–Q4 with Q1 (reference), with *P*-trend across quartiles. Basophil count was analysed as undetectable (value = 0) and detectable (value >0) due to excess zeros.

HDL-anchored ratios (MHR, NHR, PHR) exhibited the largest effect estimates in relation to incident CAD risk; leukocyte-centric ratios (*e.g*. NLR, dNLR, MLR, NMLR, SIRI, SII) were generally positively associated, albeit with smaller magnitudes on average; while platelet-anchored ratios (PLR, PWR) showed inverse associations. Specifically, each standard deviation (SD) increases in baseline PLR and PWR was associated with a 5% (95% CI = 3–6%) and 13% (95%CI = 12–15%) lower risk of CAD, respectively. In contrast, higher levels of most other inflammatory markers corresponded to modestly increased risk, with MHR demonstrating the largest association (41%; 95% CI = 37–45%) while dNLR the smallest (2%; 95% CI = 1–3%). Across all positively associated markers, the average risk increase per SD was 13.2%, although effect sizes varied considerably (**Figure 2**). Similar associations were observed when restricting CAD cases to those identified solely via hospital or death records, applying sex-specific outlier thresholds, and conducting complete-case analyses without imputation (Table S7 in the **Online Supplementary Document**). Further, the associations remained stable after adjusting for baseline use of antiplatelet agents, statins, and anticoagulants, excluding individuals with major comorbidities, and removing CAD events occurring within the first two years of follow-up (Table S8 in the **Online Supplementary Document**). E-values ranged from 1.16 to 2.16, meaning that an unmeasured confounder would need to be associated with both the exposure and the outcome by a risk ratio of at least 1.16–2.16 (conditional on measured covariates) to fully explain away the observed associations. While these findings support statistical robustness, their clinical significance should be interpreted cautiously, especially for markers with small effect sizes (*e.g*. dNLR and platelet) (**Figure 2**).

#### Threshold effects and nonlinear of inflammatory markers on CAD risk

**Figure 3:**
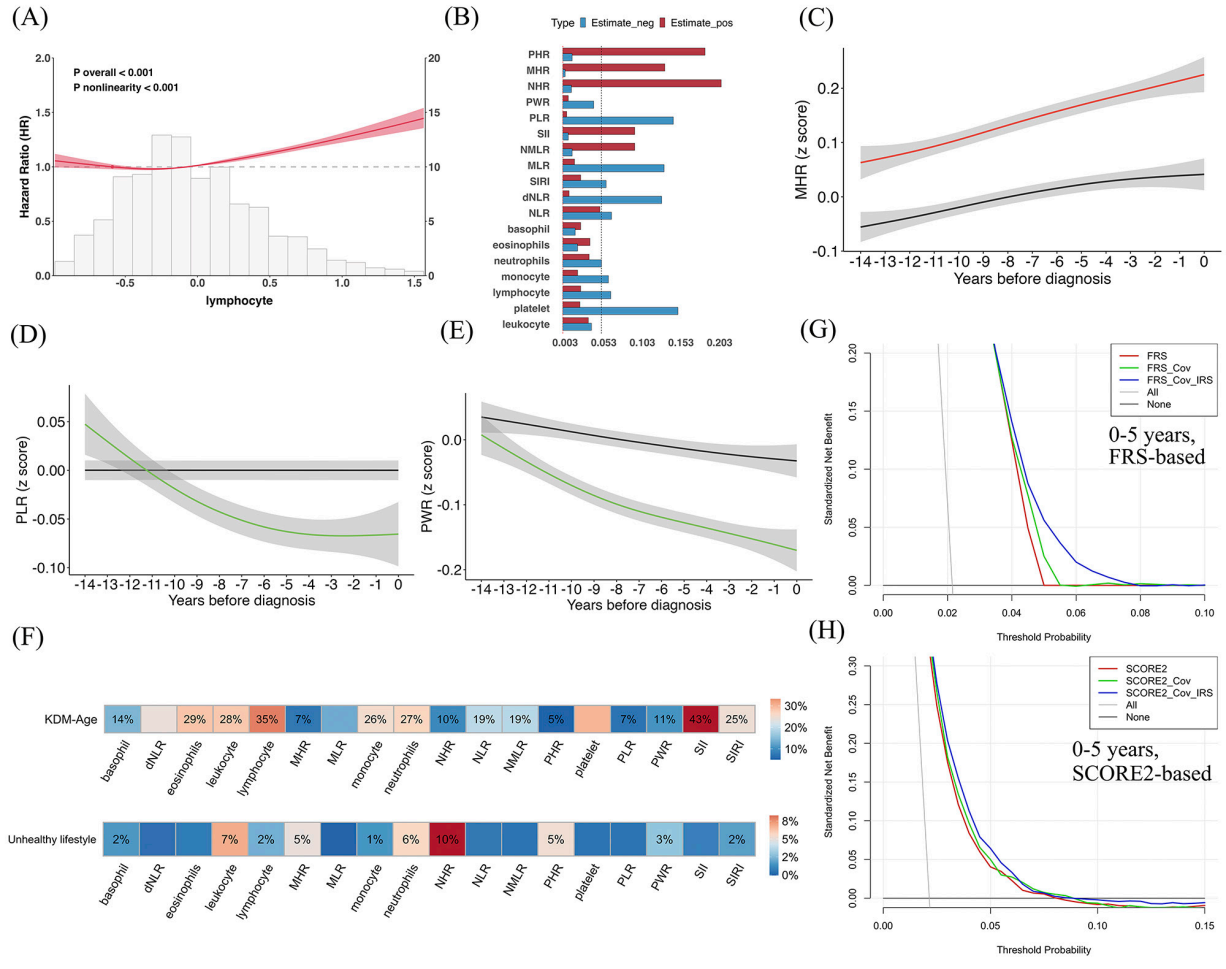
**Panel A**. Restricted cubic spline plots depicting the associations between lymphocyte and the risk of coronary artery disease (CAD). **Panel B**. Weight histogram displaying the contribution of various inflammatory markers to CAD in weighted quantile sum analyses. The red colour represents estimate of positive contribution, and blue estimate of negative contribution. **Panels C**–(**E**). Trends of monocyte-to-HDL-cholesterol ratio (MHR), platelet-to-lymphocyte ratio (PLR) and platelet-to-leukocyte ratio (PWR) in the 14 years prior to CAD diagnosis. These curves depict event-aligned population patterns rather than within-person trajectories. **Panel F**. Heatmaps of mediation proportion across two hypothesised pathways related to CAD. Colour intensity indicates the magnitude of the estimated mediation proportion, and the proportions are labelled only when the *P*_mediation_<2.8 × 10^−3^. **Panels G**–(**H**). Decision curve analysis for CAD risk prediction using Framingham risk score (FRS) and SCORE2 based models at 0 − 5 years.

Quartile-based analyses showed a dose-response relation between most inflammatory markers (except for basophil count and dNLR) and CAD risk (*P*_trend_<2.8 × 10^−3^). Briefly, compared with the lowest quartile (Q1), higher quartiles (Q2−Q4) of leukocyte, neutrophils, monocyte, SIRI, NHR, MHR and PHR were consistently associated with increased CAD risk, with the highest risk typically observed in Q4, *e.g*. for NHR (64%; 95% CI = 58–70%). Conversely, platelet-related indices (PLR and PWR) showed a decreasing trend, indicating potentially protective associations (*e.g*. for Q4 of PWR (22%; 95% CI = 19–24%) (**Figure 2**; Table S9 in the **Online Supplementary Document**). Further RCS analyses identified nonlinear risk patterns in some markers, *e.g*. lymphocyte (*P*_nonlinearity_<2.8 × 10^−3^) (**Figure 3**, panel A), indicating the complexity of inflammatory effects on CAD (Figure S2 in the **Online Supplementary Document**).

#### Stratified analyses by age, sex and CAD onset windows

Stratified analyses revealed differential effects of inflammatory markers across sex, age, and CAD onset windows. First, the effects seemed to be more evident on females than males on average (HR = 1.15 *vs*. 1.10), and four markers displayed sex-specific influences; for instance, MHR showed a stronger association with CAD risk among females (24% higher) compared to males, with similar patterns observed for NHR (10% higher), SIRI (10% higher), and PHR (5% higher) (Table S10 in the **Online Supplementary Document**). Second, eight markers showed effect differences in the age-stratified analysis; briefly, the risk effects of leukocyte, platelet count, lymphocyte, neutrophils, dNLR, NHR, and PHR were more pronounced in individuals aged ≤55 years than in those >55 years, *e.g*. 21% higher for lymphocyte, while MLR exhibited an opposite trend, 13% lower (Table S11 in the **Online Supplementary Document**). Third, to address potential violations of the PH assumption, we first tested each marker using Schoenfeld residuals and identified 11 markers with *P*-values <0.05. For these markers, we conducted stratified analyses by time to CAD onset (0 − 5 years, 5 − 10 years, and >10 years), with calendar time included as an additional covariate. In this analysis, leukocyte, neutrophils, NHR, and MHR showed stronger associations in the short-term window (0 − 5 years) compared to the long-term window (>10 years), with MHR exhibiting a 15% higher relative hazard. In contrast, PWR demonstrated a slightly stronger association with long-term CAD risk (7% greater effect in the >10 years group) (Table S12 in the **Online Supplementary Document**).

#### Weighted contribution of inflammatory markers to CAD risk

In the positive model of WQS, the top contributors were NHR (20.5%), PHR (18.4%), MHR (13.2%), SII (9.3%), and NMLR (9.3%), indicating their substantial roles in increasing CAD risk. However, in the negative model, platelet count (14.9%), PLR (14.3%), and dNLR (12.8%) showed the highest relative weights, suggesting potential protective effects at their higher levels (**Figure 3**, Panel B). These findings did not completely align with the results of Cox models, implying the varying roles of inflammatory markers in CAD and the complexity of systemic inflammation in its development.

### Inflammatory markers showed bidirectional trends prior to CAD diagnosis

In the trend analysis preceding disease onset, we observed that the levels of most inflammatory markers remained consistently higher in CAD patients compared to controls, suggesting that elevated inflammation likely preceded clinical manifestation by many years. However, platelets and their derived indices showed a gradually declined trajectory in the CAD group. Note that, the trajectories of dNLR and MLR overlapped between CAD patients and controls, which may reflect influences from individual variability or timing of measurements (Figure S3 in the **Online Supplementary Document**).

To further illustrate these dynamics, we visualised three representative markers. MHR, which had the strongest hazard in the Cox model, showed a sustained increase over the 14 years prior to CAD onset, displaying a typical pattern of cumulative risk (**Figure 3**, Panel C). In contrast, PLR and PWR demonstrated consistent downward trends from early stages, consistent with their protective impacts (**Figure 3**, Panels D–E).

### Results of the mediation analysis

KDM-age appeared to statistically mediate part of the associations between most inflammatory markers and CAD risk, with the exception of platelet, MLR, and dNLR (*P*_mediation_>2.8 × 10^−3^). Markers such as SII (estimated mediation proportion = 43%; 95% CI = 26–59%), lymphocyte (35%; 95% CI = 27–42%), and eosinophils (29%; 95% CI = 21–36%) showed relatively higher mediated proportions, suggesting that a considerable share of their observed associations with CAD may operate indirectly via accelerated aging. In contrast, markers like MHR (7%; 95% CI = 6–9%), PLR (7%; 95% CI = 4–10%), and NHR (10%; 95% CI = 8–11%) showed smaller proportions, possibly reflecting more direct or alternative pathways to CAD (**Figure 3**, Panel F).

Moreover, inflammatory markers were found to partially mediate the relationship between baseline unhealthy lifestyle factors and future CAD risk. Neutrophil-to-HDL-C ratio (NHR) (10%; 95% CI = 7–13%), leukocytes (7%; 95% CI = 5–9%), neutrophils (6%; 95% CI = 4–8%), MHR (5%; 95% CI = 3–7%), and PHR (5%; 95% CI = 3–7%) had relatively larger mediation estimates, whereas others such as dNLR, MLR, and PLR exhibited weaker or non-significant mediation effects.

### Inflammatory risk score improves CAD prediction across time windows

First, LASSO identified 10 key markers with nonzero effects. Notably, MHR and SIRI, although showing strong associations with CAD, were not selected as predictive features (Table S13 in the **Online Supplementary Document**). Then, individual IRS was calculated. Using FRS and SCORE2 as basic models, we incorporated covariates and IRS to construct prediction models with various predictors. We discovered that across all time windows (overall, 0 − 5, 5 − 10, and >10 years), the ‘FRS + Cov + IRS’ model consistently achieved the best performance across all evaluation metrics, with the most pronounced predictive accuracy and improvement observed in the short-term window (0 − 5 years) (**Table 2**, **Table 3**; Figure S4 in the **Online Supplementary Document**).

**Table 2:** Discriminative performance and reclassification improvement of FRS-based prediction models for CAD across different follow-up periods

Diagnosis time	Use FRS as the reference			
	**Model**	**AUC (95% CI)**	***P* (DeLong)**	**NRI (95% CI)**
Overall	FRS	0.705 (0.699–0.711)	\	\
	FRS + Cov	0.720 (0.713–0.726)	7.08 × 10^−32^	0.7% (0.2–1.1%)
	FRS + Cov + IRS	0.724 (0.718–0.730)	1.24 × 10^−54^	0.6% (0.1–1.2%)
0 − 5 y	FRS	0.733 (0.724–0.743)	\	\
	FRS + Cov	0.746 (0.736–0.756)	1.99 × 10^−8^	0.5% (0.1–1.0%)
	FRS + Cov + IRS	0.753 (0.743–0.763)	2.86 × 10^−19^	1.6% (1.1–2.3%)
5 − 10 y	FRS	0.704 (0.695–0.713)	\	\
	FRS + Cov	0.718 (0.709–0.728)	2.09 × 10^−13^	5.0% (4.0–6.1%)
	FRS + Cov + IRS	0.723 (0.713–0.733)	6.95 × 10^−23^	8.5% (6.9–9.9%)
>10 y	FRS	0.668 (0.657–0.678)	\	\
	FRS + Cov	0.683 (0.672–0.695)	1.43 × 10^−13^	0.4% (0.1–0.8%)
	FRS + Cov + IRS	0.686 (0.675–0.697)	7.26 × 10^−18^	0.4% (0.0–0.9%)

AUC – area under the receiver operating characteristic curve, CAD – coronary artery disease, CI – confidence interval, Cov – covariates, FRS – framingham risk score, IRS – inflammatory risk score, NRI – net reclassification improvement

**Table 3:** Discriminative performance and reclassification improvement of SCORE2-based prediction models for CAD across different follow-up periods

Diagnosis time	Use SCORE2 as the reference			
	**Model**	**AUC (95% CI)**	***P* (DeLong)**	**NRI (95% CI)**
Overall	SCORE2	0.699 (0.693–0.706)	\	\
	SCORE2 + Cov	0.705 (0.698–0.711)	2.70 × 10^−3^	2.1% (1.1–3.1%)
	SCORE2 + Cov + IRS	0.707 (0.701–0.714)	1.99 × 10^−4^	3.1% (2.0–4.2%)
0 − 5 y	SCORE2	0.722 (0.711–0.734)	\	\
	SCORE2 + Cov	0.727 (0.716–0.739)	0.09	0.6% (-0.1, 1.3%)
	SCORE2 + Cov + IRS	0.729 (0.718–0.741)	0.06	0.9% (0.0–2.1%)
5 − 10 y	SCORE2	0.693 (0.683–0.703)	\	\
	SCORE2 + Cov	0.698 (0.688–0.709)	0.10	1.3% (0.5–2.3%)
	SCORE2 + Cov + IRS	0.701 (0.690–0.711)	4.26 × 10^−2^	2.7% (1.7–3.6%)
>10 y	SCORE2	0.672 (0.661–0.683)	\	\
	SCORE2 + Cov	0.676 (0.665–0.688)	0.25	0.6% (0.0–1.3%)
	SCORE2 + Cov + IRS	0.679 (0.667–0.690)	0.11	1.1% (0.4–1.8%)

AUC – area under the receiver operating characteristic curve, CAD – coronary artery disease, Cov – covariates, CI – confidence interval, IRS – inflammatory risk score, NRI – net reclassification improvement

Specifically, for FRS-based models, the AUC significantly increased from 0.733 (95 CI% = 0.724–0.743) to 0.746 (95 CI% = 0.736–0.756) for the short-term CAD risk after adding covariates, representing a 1.8% (*P*_DeLong_ = 1.99 × 10^−8^) improvement, and further rose to 0.753 (95 CI% = 0.743–0.763) with the inclusion of IRS, yielding a total gain of 2.7% (*P*_DeLong_ = 2.86 × 10^−19^). NRI analysis indicated a 1.6% (95 CI% = 1.1–2.3%) improvement in reclassification for the integrated model compared with FRS alone, driven by 35 of 1700 CAD cases (2.1%) being reclassified from below to above the 7.5% risk threshold, at the cost of 350 of 77 473 non-cases (0.5%) also moving into the higher-risk category, with no reclassification from high to low risk in either group (**Table 2**; Table S14 in the **Online Supplementary Document**). Decision curve analyses (DCA) further confirmed that the ‘FRS + Cov + IRS’ model offered greater clinical net benefit within the 5–10% risk threshold range, with an approximate gain about 5% (**Figure 3**, Panel G). A similar pattern was observed for models based on SCORE2. Although their overall AUCs were slightly lower than those of FRS-based models, the integration of IRS consistently improved model discrimination. Further, the ‘SCORE2 + Cov + IRS’ model provided modest yet stable net benefit across a broader threshold range (5–15%) (**Figure 3**, Panel H).

The other prediction windows (5 − 10 and >10 years) also favoured the IRS-integrated models, although the magnitude of predictive improvement was somewhat attenuated compared to the short-term setting (**Table 2**, **Table 3**; Figure S4 in the **Online Supplementary Document**).

## DISCUSSION

### Summary results of this study

In this large prospective cohort of 475 134 participants, we examined associations between 18 CBC-based inflammatory markers and incident CAD. Many markers showed statistically significant associations, with MHR, NHR, and SIRI generally demonstrating the strongest positive relationships, whereas PLR and PWR tended to be inversely associated. Group-level differences in several markers were observable up to ~ 10 years before CAD diagnosis. Signals suggesting that accelerated aging may mediate the relationship between inflammation and CAD, and that inflammatory markers may partly mediate the effect of unhealthy lifestyle on CAD, are exploratory and of modest magnitude. Finally, an integrated IRS incorporating key markers yielded a modest improvement in short-term (0 − 5 years) risk discrimination beyond traditional predictors in internal validation.

### Inflammatory markers play different roles in CAD

#### Family-wise interpretation of inflammatory markers and CAD risk

The HDL-anchored ratios (MHR, NHR, PHR) showed the strongest positive associations with incident CAD; leukocyte-centric ratios (*e.g*. NLR, dNLR, MLR, NMLR, SIRI, SII) were generally positively associated with smaller average effects; and platelet-anchored ratios (PLR, PWR) tended to be inversely associated and declined over time. Taken together, these patterns suggest the presence of distinct biological axes, including lipid-linked inflammatory imbalance, innate-adaptive immune shifts, and thrombo-inflammatory or platelet-related dynamics, rather than a single unified pathway. Group-level differences were observable years before diagnosis, but these temporal trends should not be interpreted as causal.

Markers such as MHR, which integrate inflammatory and lipid-related information, may function as high sensitivity but low specificity indicators of systemic inflammatory activation during the early stages of disease. Their elevations may reflect not only CAD specific processes but also non-specific responses to infections, psychological stress, or metabolic disturbances [36]. It should be noted that MHR shows a strong positive correlation with CAD and has not been selected as a predictive factor by LASSO. Statistically, LASSO favours a sparse set of less-correlated, most predictive features; when composites are highly correlated with their components or with other ratios, their apparent single-marker signal can be absorbed by more stable or less collinear variables [35]. Consequently, biologically relevant markers may be excluded without contradicting their association in marginal models. This family-wise framing also helps contextualise earlier reports that evaluated single markers in isolation and may have over-stated independent effects or predictive utility [15,37,38].

Within the platelet family, PLR and PWR consistently showed inverse associations with incident CAD across Cox and WQS models and exhibited long-term declines prior to diagnosis. This cross-method analysis supports the robustness of the inverse direction in our data. Although this pattern appears counter to the conventional expectation that higher platelet levels enhance thrombosis and thereby increase CAD risk [39–41]. During chronic vascular inflammation, platelets may undergo persistent activation and consumption through subclinical thrombo-inflammatory processes, including endothelial repair, microthrombus formation, and platelet-leukocyte interactions along the vascular wall. This can result in lower circulating platelet counts despite elevated functional activity [42]. Thus, a reduced platelet count or a lower platelet-anchored ratio when denominators rise does not necessarily imply reduced thrombogenicity, but may reflect advanced or active disease biology [43]. In short, count-based indices and functional thrombogenic potential need not move in the same direction.

For leukocyte-centric ratios such as dNLR or MLR, positive single-marker Cox associations co-existed with relatively larger negative weights in the WQS mixture. First, WQS imposes sign constraints and aggregates correlated markers, so weights capture shared variation within and across families rather than mirroring marginal HRs one-to-one. Second, nonlinear effects (as seen with the RCS analyses) and network interactions in inflammatory pathways [44] can cause a composite to contribute risk in Cox while receiving down weighted or opposite signed influence in a constrained mixture when correlated partners already account for the shared signal. So that the mixture-model weights and single-marker estimates may diverge without negating either.

#### Stratified, nonlinear, and quartile-based analyses revealed distinct inflammatory risk profiles

Stratified analyses revealed that the impact of certain inflammatory markers on CAD risk varies by sex, age, and onset time, with stronger associations in females, younger individuals, and short-term-onset cases. The sex differences may be due to hormonal and immune system variations, as women generally have stronger immune responses and greater sensitivity to inflammatory stimuli than men [45]. A study in postmenopausal women has shown that combined hormone therapy (exogenous oestrogen and progestin) can significantly increase the risk of CAD [46]. In younger people or those with short-term CAD, the inflammatory response is more pronounced, and marker levels may indicate early vascular damage and plaque formation [47]. In older individuals or those with long-term CAD, aging-related metabolic changes and chronic immune issues, such as reduced T cell immunity and mitochondrial function, play a more significant role than acute inflammation [48,49]. Further, the RCS analysis showed a nonlinear link between markers like NHR and MHR and CAD risk, and quartile analysis indicated a clear dose-response relationship, with higher quartiles linked to greater CAD risk. This highlights those inflammatory markers impact CAD risk differently based on exposure levels, emphasising the need for early intervention, such as anti-inflammatory therapies [3].

### Aging may mediate the impact of systemic inflammation on CAD

Our findings indicate that inflammation may influence CAD risk through several potential intermediary pathways. In the exploratory mediation framework ‘Inflammation → Aging → CAD’, SII, lymphocytes, and eosinophils exhibited comparatively higher mediation proportions (ranging from 29% to 43%). This statistical pattern is consistent with the inflammaging hypothesis [20], which posits that chronic low-grade inflammation accelerates biological aging via immune dysfunction and cellular senescence. Supporting this concept, recent studies have reported that certain immune regulators, such as IgG, may contribute to age-related tissue dysfunction [50]. However, these mechanisms require further confirmation using longitudinal data with repeated biomarker assessments.

In the ‘Unhealthy lifestyle → Inflammation → CAD’ model, markers such as NHR and leukocytes showed smaller mediated proportions (7–10%). While statistically significant in this large sample, these modest estimates imply that inflammation may function as one of several modulatory pathways linking lifestyle and cardiovascular risk. Previous studies have suggested that inflammatory responses might amplify lifestyle-related CAD susceptibility [21], but the extent of this interaction remains to be fully elucidated. We caution against over-interpreting these findings in causal or mechanistic terms.

### IRS has consistently improved the performance of classic cardiovascular prediction models

In this work, the proposed IRS offers a practical method to quantify inflammation and improve disease prediction, significantly and consistently enhancing the FRS and SCORE2 models' accuracy across multiple time windows, especially in short-term (0 − 5 years) CAD risk prediction, with a notable increase in discrimination (AUC = 2.7%), NRI (1.6%) and clinical net benefit ( ~ 5%). Given the large burden of CAD [1], even modest gains in net benefit can translate into substantially earlier identification and intervention opportunities for a considerable number of high-risk individuals. Compared to traditional models relying on single or limited markers [15,51], IRS integrates a diverse set of inflammatory signals, offering a more stable and comprehensive assessment of inflammation. Further, IRS relies solely on routine CBC parameters, making it affordable and accessible. This suitability enhances early warning and intervention in primary care. However, it should be noted that the generalisability of the IRS primarily applies to populations similar to the UK Biobank. When applied to more diverse populations, local recalibration and standardisation are recommended prior to implementation.

### Limitations of the present study and further research

To our knowledge, this study is the first large-scale and systematic exploration of the impact and prediction of inflammatory markers on CAD; nevertheless, there are some limitations needed to mention. First, our findings may not apply to much younger populations as the samples analysed were predominantly composed of middle-aged to older adults. Second, static inflammatory markers do not fully reflect long-term changes, limiting insights into CAD risk progression over time [52]. Third, while enhancing statistical efficiency and aligning with real-world diagnostic practices, the composite CAD endpoint may dilute subtype-specific etiologic signals. Future research should validate and recalibrate our findings using more refined and clinically adjudicated outcomes. Fourth, although the IRS demonstrated added predictive value, it has not yet undergone broad external validation, and further studies in independent cohorts are warranted to confirm its generalisability and clinical utility. Finally, the observed associations and mediation pathways were derived from observational data, which precludes strong causal inference. Experimental or interventional studies are needed to further clarify the causal role of systemic inflammation in CAD pathogenesis.

## CONCLUSIONS

In this large prospective cohort, multiple routine inflammatory markers showed consistent associations with CAD risk. An integrated IRS modestly improved short-term risk discrimination beyond established predictors in internal validation. Signals that accelerated aging may partially mediate these associations should be interpreted cautiously given the strong assumptions and small-to-moderate effect sizes. Future work should prioritise external validation in diverse populations and determine clinical utility.

## Additional material


Online Supplementary Document

## References

[1] GBD 2017 Causes of Death Collaborators Global, regional, and national age-sex-specific mortality for 282 causes of death in 195 countries and territories, 1980–2017: a systematic analysis for the Global Burden of Disease Study 2017. Lancet. 2018;392:1736−88. 10.1016/S0140-6736(18)32203-7 30496103 PMC6227606

[2] Global Cardiovascular Risk Consortium Magnussen C Ojeda FM Leong DP Alegre-Diaz J Amouyel P Global Effect of Modifiable Risk Factors on Cardiovascular Disease and Mortality. N Engl J Med. 2023;389:1273−85. 10.1056/NEJMoa2206916 37632466 PMC10589462

[3] Zakynthinos E Pappa N Inflammatory biomarkers in coronary artery disease. J Cardiol. 2009;53:317−33. 10.1016/j.jjcc.2008.12.007 19477372

[4] Medina-Leyte DJ Zepeda-García O Domínguez-Pérez M González-Garrido A Villarreal-Molina T Jacobo-Albavera L Endothelial Dysfunction, Inflammation and Coronary Artery Disease: Potential Biomarkers and Promising Therapeutical Approaches. Int J Mol Sci. 2021;22:3850. 10.3390/ijms22083850 33917744 PMC8068178

[5] Urbanowicz T Olasińska-Wiśniewska A Michalak M Komosa A Filipiak KJ Uruski P Predictive role of monocyte count for significant coronary artery disease identification in patients with stable coronary artery disease. Cardiol J. 2024;31:722−30. 10.5603/cj.95131 38149491 PMC11544409

[6] Chen S Zhang S Luan H Zeng X Li Y Yuan H Correlation Between Extended Leukocyte Differential Count and Coronary Artery Disease. J Cardiovasc Pharmacol. 2018;71:359−66. 10.1097/FJC.0000000000000582 29668531

[7] Lee CD Folsom AR Nieto FJ Chambless LE Shahar E Wolfe DA White blood cell count and incidence of coronary heart disease and ischemic stroke and mortality from cardiovascular disease in African-American and White men and women: atherosclerosis risk in communities study. Am J Epidemiol. 2001;154:758−64. 10.1093/aje/154.8.758 11590089

[8] In Het Panhuis W Schönke M Modder M Tom HE Lalai RA Pronk ACM Time-restricted feeding attenuates hypercholesterolaemia and atherosclerosis development during circadian disturbance in APOE*3-Leiden.CETP mice. EBioMedicine. 2023;93:104680. 10.1016/j.ebiom.2023.104680 37356205 PMC10320519

[9] Raggi P Genest J Giles JT Rayner KJ Dwivedi G Beanlands RS Role of inflammation in the pathogenesis of atherosclerosis and therapeutic interventions. Atherosclerosis. 2018;276:98−108. 10.1016/j.atherosclerosis.2018.07.014 30055326

[10] Passos LSA Lupieri A Becker-Greene D Aikawa E Innate and adaptive immunity in cardiovascular calcification. Atherosclerosis. 2020;306:59−67. 10.1016/j.atherosclerosis.2020.02.016 32222287 PMC7483874

[11] Dziedzic EA Gąsior JS Tuzimek A Paleczny J Junka A Dąbrowski M Investigation of the Associations of Novel Inflammatory Biomarkers-Systemic Inflammatory Index (SII) and Systemic Inflammatory Response Index (SIRI)-With the Severity of Coronary Artery Disease and Acute Coronary Syndrome Occurrence. Int J Mol Sci. 2022;23:9553. 10.3390/ijms23179553 36076952 PMC9455822

[12] Zhao Z Zhang X Sun T Huang X Ma M Yang S Prognostic value of systemic immune-inflammation index in CAD patients: Systematic review and meta-analyses. Eur J Clin Invest. 2024;54:e14100. 10.1111/eci.14100 37776036

[13] Pan Q Zhang W Li X Chen Z Yang Y Wang G Sex Difference in the Association Between Neutrophil to Lymphocyte Ratio and Severity of Coronary Artery Disease. Angiology. 2022;73:470−7. 10.1177/00033197211070884 35129378

[14] Soran H Hama S Yadav R Durrington PN HDL functionality. Curr Opin Lipidol. 2012;23:353−66. 10.1097/MOL.0b013e328355ca25 22732521

[15] Manoochehri H Gheitasi R Pourjafar M Amini R Yazdi A Investigating the relationship between the severity of coronary artery disease and inflammatory factors of MHR, PHR, NHR, and IL-25. Med J Islam Repub Iran. 2021;35:85. 10.47176/mjiri.35.85 34291009 PMC8285545

[16] Miedema MD Dardari ZA Nasir K Blankstein R Knickelbine T Oberembt S Association of Coronary Artery Calcium With Long-term, Cause-Specific Mortality Among Young Adults. JAMA Netw Open. 2019;2:e197440. 10.1001/jamanetworkopen.2019.7440 31322693 PMC6646982

[17] D’Agostino RB Sr Vasan RS Pencina MJ Wolf PA Cobain M Massaro JM General cardiovascular risk profile for use in primary care: the Framingham Heart Study. Circulation. 2008;117:743−53. 10.1161/CIRCULATIONAHA.107.699579 18212285

[18] SCORE2 working group and ESC Cardiovascular risk collaboration SCORE2 risk prediction algorithms: new models to estimate 10-year risk of cardiovascular disease in Europe. Eur Heart J. 2021;42:2439−54. 10.1093/eurheartj/ehab309 34120177 PMC8248998

[19] Sudlow C Gallacher J Allen N Beral V Burton P Danesh J UK Biobank: An Open Access Resource for Identifying the Causes of a Wide Range of Complex Diseases of Middle and Old Age. PLoS Med. 2015;12:e1001779. 10.1371/journal.pmed.1001779 25826379 PMC4380465

[20] Li X Li C Zhang W Wang Y Qian P Huang H Inflammation and aging: signaling pathways and intervention therapies. Signal Transduct Target Ther. 2023;8:239. 10.1038/s41392-023-01502-8 37291105 PMC10248351

[21] Blaum C Brunner FJ Kröger F Braetz J Lorenz T Goßling A Modifiable lifestyle risk factors and C-reactive protein in patients with coronary artery disease: Implications for an anti-inflammatory treatment target population. Eur J Prev Cardiol. 2021;28:152−8. 10.1177/2047487319885458 33838040

[22] Rudan I Song P Adeloye D Campbell H Journal of Global Health’s Guidelines for Reporting Analyses of Big Data Repositories Open to the Public (GRABDROP): preventing ‘paper mills’, duplicate publications, misuse of statistical inference, and inappropriate use of artificial intelligence. J Glob Health. 2025;15:01004. 10.7189/jogh.15.01004 40587200 PMC12208284

[23] Jin Z Wu Q Chen S Gao J Li X Zhang X The Associations of Two Novel Inflammation Indexes, SII and SIRI with the Risks for Cardiovascular Diseases and All-Cause Mortality: A Ten-Year Follow-Up Study in 85,154 Individuals. J Inflamm Res. 2021;14:131−40. 10.2147/JIR.S283835 33500649 PMC7822090

[24] Kivimäki M Nyberg ST Fransson EI Heikkilä K Alfredsson L Casini A Associations of job strain and lifestyle risk factors with risk of coronary artery disease: a meta-analysis of individual participant data. CMAJ. 2013;185:763−9. 10.1503/cmaj.121735 23670152 PMC3680555

[25] Jousilahti P Vartiainen E Tuomilehto J Puska P Sex, age, cardiovascular risk factors, and coronary heart disease: a prospective follow-up study of 14 786 middle-aged men and women in Finland. Circulation. 1999;99:1165−72. 10.1161/01.CIR.99.9.1165 10069784

[26] VanderWeele TJ Ding P Sensitivity Analysis in Observational Research: Introducing the E-Value. Ann Intern Med. 2017;167:268−74. 10.7326/M16-2607 28693043

[27] Williams RR Hunt SC Heiss G Province MA Bensen JT Higgins M Usefulness of cardiovascular family history data for population-based preventive medicine and medical research (the Health Family Tree Study and the NHLBI Family Heart Study). Am J Cardiol. 2001;87:129−35. 10.1016/S0002-9149(00)01303-5 11152826

[28] Yahagi K Davis HR Arbustini E Virmani R Sex differences in coronary artery disease: pathological observations. Atherosclerosis. 2015;239:260−7. 10.1016/j.atherosclerosis.2015.01.017 25634157

[29] Carrico C Gennings C Wheeler DC Factor-Litvak P Characterization of Weighted Quantile Sum Regression for Highly Correlated Data in a Risk Analysis Setting. J Agric Biol Environ Stat. 2015;20:100−20. 10.1007/s13253-014-0180-3 30505142 PMC6261506

[30] Cleveland WS Devlin SJ Locally Weighted Regression: An Approach to Regression Analysis by Local Fitting. J Am Stat Assoc. 1988;83:596−610. 10.1080/01621459.1988.10478639

[31] Abadie A Imbens GW Matching on the estimated propensity score. Econometrica. 2016;84:781−807. 10.3982/ECTA11293

[32] Klemera P Doubal S A new approach to the concept and computation of biological age. Mech Ageing Dev. 2006;127:240−8. 10.1016/j.mad.2005.10.004 16318865

[33] Zhang YB Chen C Pan XF Guo J Li Y Franco OH Associations of healthy lifestyle and socioeconomic status with mortality and incident cardiovascular disease: two prospective cohort studies. BMJ. 2021;373:n604. 10.1136/bmj.n604 33853828 PMC8044922

[34] Zhang N Liu X Wang L Zhang Y Xiang Y Cai J Lifestyle factors and their relative contributions to longitudinal progression of cardio-renal-metabolic multimorbidity: a prospective cohort study. Cardiovasc Diabetol. 2024;23:265. 10.1186/s12933-024-02347-3 39026309 PMC11264843

[35] Ranstam J Cook JA LASSO regression. Br J Surg. 2018;105:1348. 10.1002/bjs.10895

[36] Libby P The changing landscape of atherosclerosis. Nature. 2021;592:524−33. 10.1038/s41586-021-03392-8 33883728

[37] Yilu Z Zhanglong W Fanke H Jing G Yue W Yuwen C The progression of non-culprit coronary lesion is related to higher SII, SIRI, and PIV in patients with ACS. Medicine (Baltimore). 2024;103:e41094. 10.1097/MD.0000000000041094 39969298 PMC11688051

[38] Korkmaz A Demir M Unal S Yildiz A Ozyazgan B Demirtas B Monocyte-to-high density lipoprotein ratio (MHR) can predict the significance of angiographically intermediate coronary lesions. International Journal of the Cardiovascular Academy. 2017;3:16−20. 10.1016/j.ijcac.2017.05.008

[39] Akboga MK Canpolat U Yayla C Ozcan F Ozeke O Topaloglu S Association of Platelet to Lymphocyte Ratio With Inflammation and Severity of Coronary Atherosclerosis in Patients With Stable Coronary Artery Disease. Angiology. 2016;67:89−95. 10.1177/0003319715583186 25922197

[40] Qiu Z Jiang Y Jiang X Yang R Wu Y Xu Y Relationship Between Platelet to Lymphocyte Ratio and Stable Coronary Artery Disease: Meta-Analysis of Observational Studies. Angiology. 2020;71:909−15. 10.1177/0003319720943810 32720814

[41] Lee YSG Baradi A Peverelle M Sultani R Adams H Garlick J Usefulness of Platelet-to-Lymphocyte Ratio to Predict Long-Term All-Cause Mortality in Patients at High Risk of Coronary Artery Disease Who Underwent Coronary Angiography. Am J Cardiol. 2018;121:1021−6. 10.1016/j.amjcard.2018.01.018 29606325

[42] Koupenova M Clancy L Corkrey HA Freedman JE Circulating Platelets as Mediators of Immunity, Inflammation, and Thrombosis. Circ Res. 2018;122:337−51. 10.1161/CIRCRESAHA.117.310795 29348254 PMC5777300

[43] Jasani J Modi M Vaishnani H Gharia B Shah Y Patel D Evaluation of platelet count and platelet indices in patients with coronary artery disease. IJBAR. 2014;5:553−5. 10.7439/ijbar.v5i11.978

[44] Libby P Hansson GK Inflammation and immunity in diseases of the arterial tree: players and layers. Circ Res. 2015;116:307−11. 10.1161/CIRCRESAHA.116.301313 25593275 PMC4299915

[45] Lau ES Paniagua SM Guseh JS Bhambhani V Zanni MV Courchesne P Sex Differences in Circulating Biomarkers of Cardiovascular Disease. J Am Coll Cardiol. 2019;74:1543−53. 10.1016/j.jacc.2019.06.077 31537263 PMC6756178

[46] Manson JE Hsia J Johnson KC Rossouw JE Assaf AR Lasser NL Estrogen plus progestin and the risk of coronary heart disease. N Engl J Med. 2003;349:523−34. 10.1056/NEJMoa030808 12904517

[47] Attiq A Afzal S Ahmad W Kandeel M Hegemony of inflammation in atherosclerosis and coronary artery disease. Eur J Pharmacol. 2024;966:176338. 10.1016/j.ejphar.2024.176338 38242225

[48] Franceschi C Campisi J Chronic inflammation (inflammaging) and its potential contribution to age-associated diseases. J Gerontol A Biol Sci Med Sci. 2014;69 Suppl 1:S4−9. 10.1093/gerona/glu057 24833586

[49] Han S Georgiev P Ringel AE Sharpe AH Haigis MC Age-associated remodeling of T cell immunity and metabolism. Cell Metab. 2023;35:36−55. 10.1016/j.cmet.2022.11.005 36473467 PMC10799654

[50] Yu L Wan Q Liu Q Fan Y Zhou Q Skowronski AA IgG is an aging factor that drives adipose tissue fibrosis and metabolic decline. Cell Metab. 2024;36:793−807.e5. 10.1016/j.cmet.2024.01.015 38378001 PMC11070064

[51] Yang YL Wu CH Hsu PF Chen SC Huang SS Chan WL Systemic immune-inflammation index (SII) predicted clinical outcome in patients with coronary artery disease. Eur J Clin Invest. 2020;50:e13230. 10.1111/eci.13230 32291748

[52] Nash SD Cruickshanks KJ Klein R Klein BE Nieto FJ Chappell R Long-term variability of inflammatory markers and associated factors in a population-based cohort. J Am Geriatr Soc. 2013;61:1269−76. 10.1111/jgs.12382 23889670 PMC3743937

