# Erratum: Zhang H, Liu Y, Yan Y, Qi J, Lin H, Jiang Y, Wang X, Cao H, Jiang Z, Zhang S, Wang T, Xu Y, Song W, Wang K, Zheng C, Zeng P. Unravelling the role of inflammatory markers in coronary artery disease risk via association, mediation and prediction analyses. J Glob Health. 2026;16:04060.

**DOI:** 10.7189/jogh.16.1604060err1

**Published:** 2026-02-20

**Authors:** Journal of Global Health Editorial Team



Following the publication of the manuscript ‘Unravelling the role of inflammatory markers in coronary artery disease risk via association, mediation and prediction analyses’ on 13 February 2026, several errors were identified in the published article. Three figures were included in error during the production process and were not part of the final accepted manuscript. The figures in the online version of the article were correct; these errors affected the PDF version only. In addition, two subheadings were incorrect. On pages 7 and 8, the two subheadings were mistakenly published with the identical title ‘Threshold effects and nonlinear of inflammatory markers on CAD risk’.

The incorrect figures have been removed and replaced with the correct figures in the PDF version of the article, and the duplicated subheadings have been corrected in both the PDF and online versions. The editorial team regrets this error, takes full responsibility, and apologises for any inconvenience it may have caused to the readers or the authors.

These corrections have been made to the PDF version of the manuscript on 20 February 2026, following the authors’ notice on 15 February 2026.

