# Supplementary material for: Unravelling the role of inflammatory markers in coronary artery disease risk via association, mediation and prediction analyses
Source: J Glob Health. 2026 Feb 13;16:04060. doi: 10.7189/jogh.16.04060 (PMC12903186; doi:10.7189/jogh.16.04060)
Supplement: Online Supplementary Document [file jogh-16-04060-s001.pdf]

Supplement to: Zhang H, Liu Y, Yan Y, Qi J, Lin H, Jiang Y, Wang X, Cao H, Jiang Z, Zhang S, Wang T, Xu Y, Song W, Wang K, Zheng C, Zeng P. Unravelling the role of inflammatory markers in coronary artery disease risk via association, mediation and prediction analyses. J Glob Health. 2026;16:04060.

Table of contents

Calculation of KDM-BA age
Figure S1. The heatmap displays the correlation and clustering between inflammatory markers.
Figure S2. Restricted cubic spline plots depicting the associations between inflammatory markers and the risk of coronary artery disease (CAD).
Figure S3. Trends of inflammatory markers in the 14 years prior to coronary artery disease (CAD) diagnosis.
Figure S4. Decision curve analysis (DCA) for CAD risk prediction using FRS and SCORE2-based models at different time windows (overall, 0-5 years, 5-10 years, and >10 years).
Table S1. Outline of JoGH guideline items.
Table S2. Definitions of the exposure, outcome, and covariates from the UK Biobank.
Table S3. Sample inclusion before and after exclusion of extreme inflammatory marker values.
Table S4. Missingness patterns for clinical covariates in the analysis cohort.
Table S5. STROBE Statement—Checklist of items that should be included in reports of cohort studies
Table S6. Baseline characteristics of the participants analyzed in our study.
Table S7. Sensitivity analyses of CAD risk models with inflammatory markers: excluding self-reported outcomes, sex-specific outlier handling and complete-case analysis.
Table S8. Sensitivity analyses of CAD risk models with inflammatory markers: medication adjustment, disease exclusions, and removal of early events.
Table S9. Associations between inflammatory markers and CAD risk across quartiles.
Table S10. Relation between inflammatory markers and CAD risk in the baseline population stratified by sex.
Table S11. Relation between inflammatory markers and CAD risk in the baseline population stratified by age.
Table S12. Time-stratified Cox regression and proportional hazards (PH) assumption testing for inflammatory markers and CAD risk.
Table S13. Key inflammation variables and their estimated coefficient in the LASSO-derived inflammatory score.
Table S14. Net Reclassification Improvement (NRI) and number of reclassified individuals across models and time windows at the 7.5% risk threshold.
References

Calculation of KDM-BA age

The participants' KDM-BA age was calculated based on actual age and nine clinical blood biomarkers, including forced expiratory volume in one second (FEV1), systolic blood pressure, albumin, alkaline phosphatase, blood urea nitrogen, creatinine, C-reactive protein, glycated hemoglobin, and total cholesterol, and chronological age at baseline with the algorithm proposed by [1]. The formula of KDM-BA is

$$\text{KDM-BA} = \frac{\sum_{j=1}^{n}\left(x_j - q_j\right)\left(\frac{k_j}{s_j^2}\right) + \frac{CA}{s_{BA}^2}}{\sum_{j=1}^{n}\left(\frac{k_j}{s_j}\right)^2 + \frac{1}{s_{BA}^2}}$$

where *x* represents the jth biomarker measure; *CA* represents chronological age; *q*, *k*, and *s* represent parameters when regressed the jth biomarker on *CA*; *sBA* represents a scaling factor equal to the square root of the variance in chronological age explained by the biomarker set.

We removed the 1% and 99% extreme values of KDM-BA age. KDM-BA age acceleration is defined as the residuals of KDM-BA age adjusted for chronological age using a general linear model, where larger residuals indicate more pronounced age acceleration [2]. In this study, KDM-BA age residuals were applied in our analyses for the mediation.

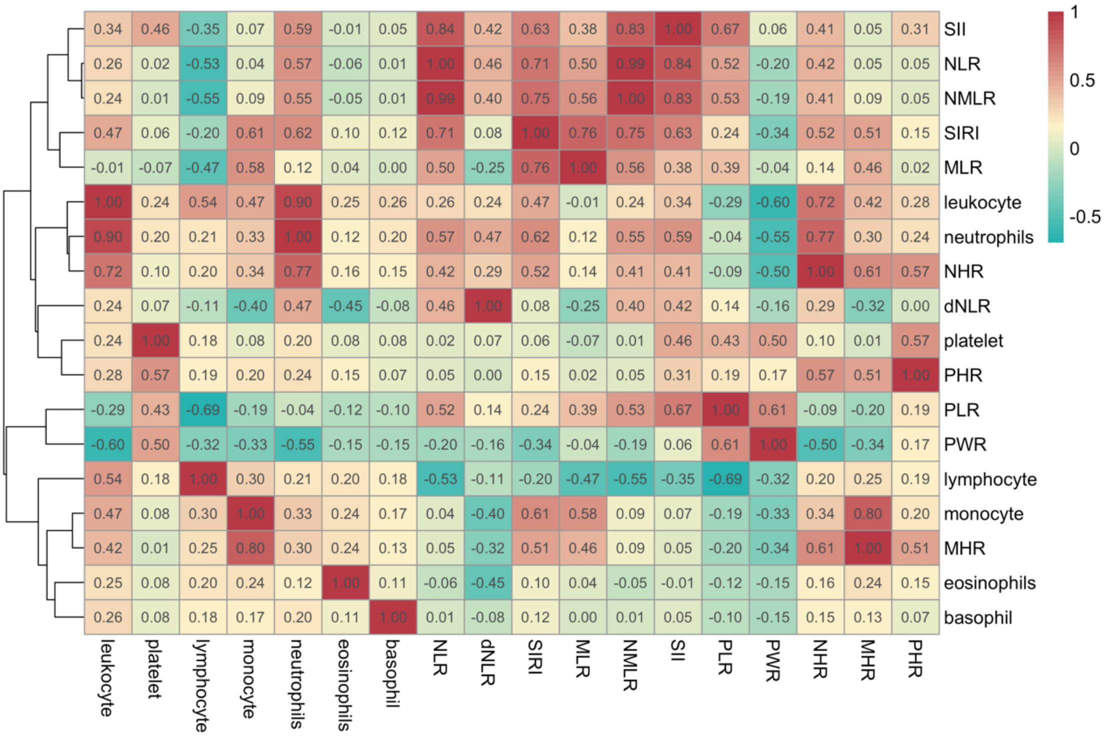

Figure S1. The heatmap displays the correlation and clustering between inflammatory markers. The red color represents positive correlation, and blue color represents negative correlation; the depth of color is directly proportional to the absolute value of the correlation coefficient. The length of tree branches reflects the similarity between markers, while short branches indicate high correlation.

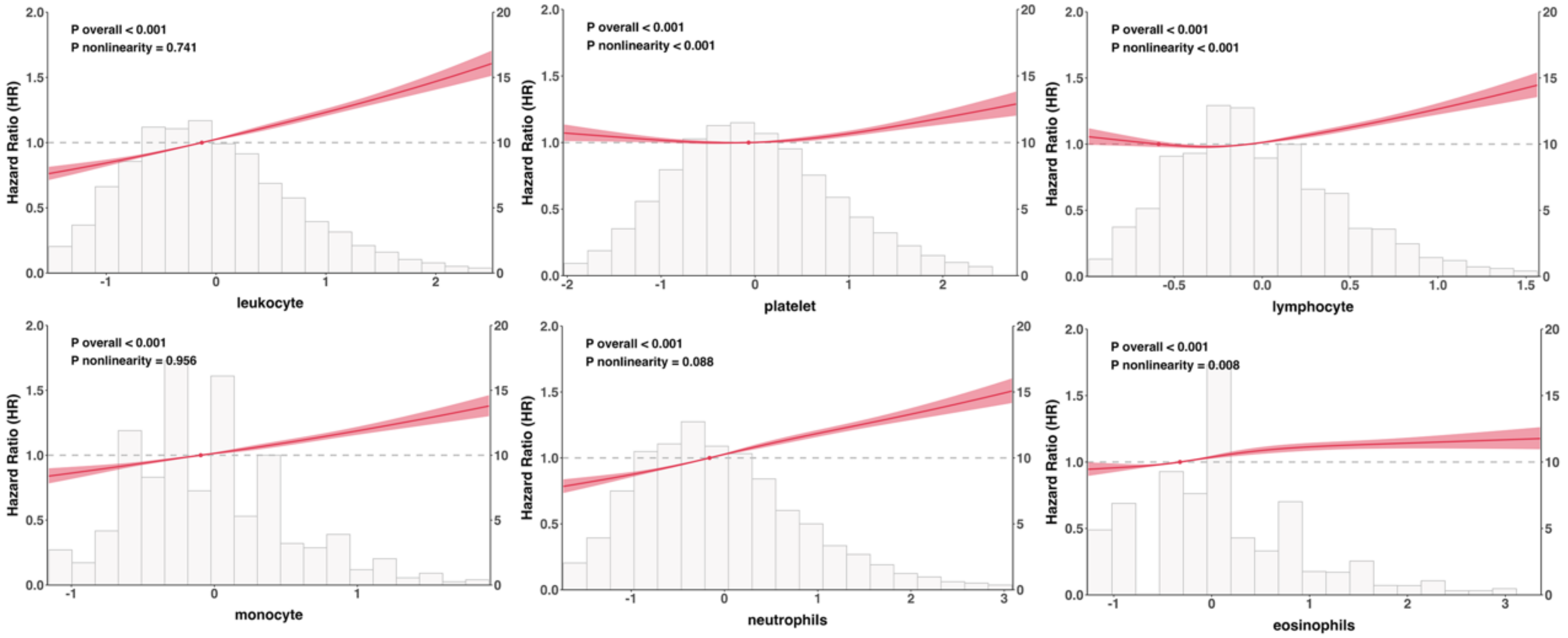
P overall < 0.001
P nonlinearity = 0.741
Hazard Ratio (HR)
leukocyte
P overall < 0.001
P nonlinearity < 0.001
Hazard Ratio (HR)
platelet
P overall < 0.001
P nonlinearity < 0.001
Hazard Ratio (HR)
lymphocyte
P overall < 0.001
P nonlinearity = 0.956
Hazard Ratio (HR)
monocyte
P overall < 0.001
P nonlinearity = 0.088
Hazard Ratio (HR)
neutrophils
P overall < 0.001
P nonlinearity = 0.008
Hazard Ratio (HR)
eosinophils

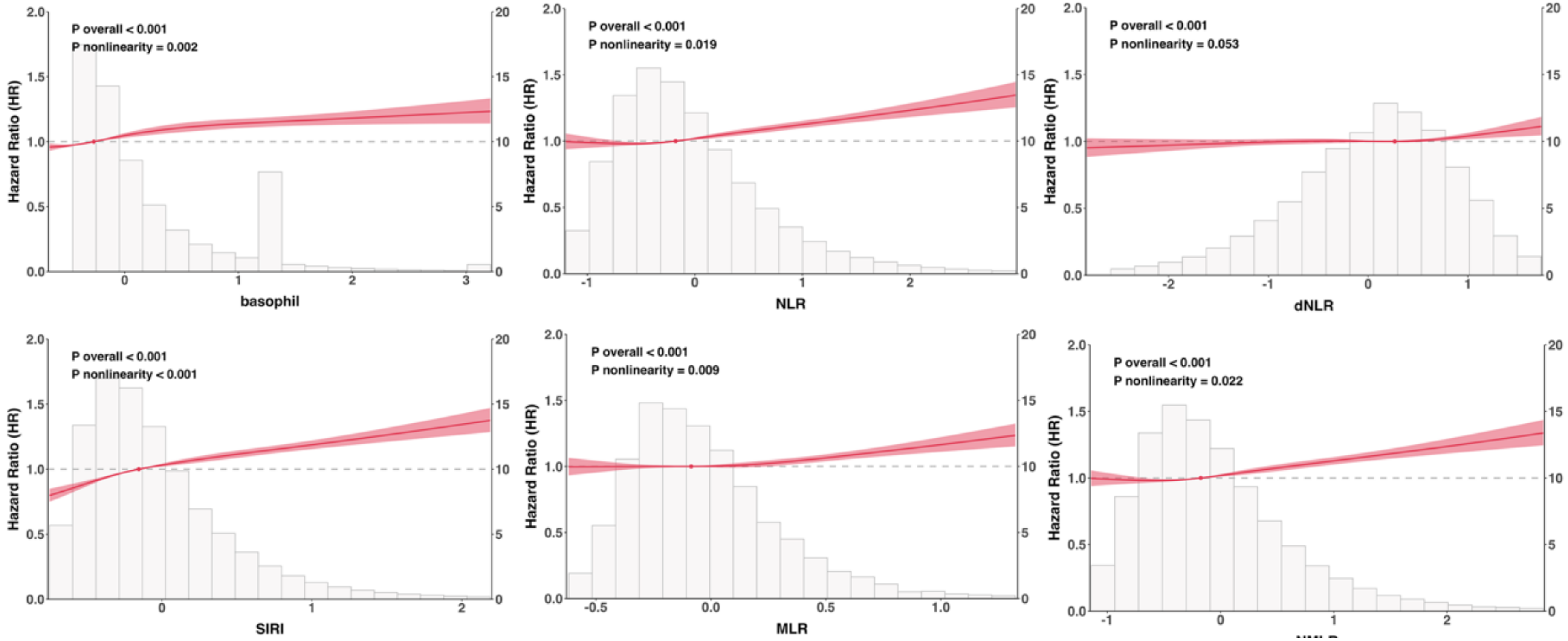
P overall < 0.001
P nonlinearity = 0.002
Hazard Ratio (HR)
basophil
P overall < 0.001
P nonlinearity = 0.019
Hazard Ratio (HR)
NLR
P overall < 0.001
P nonlinearity = 0.053
Hazard Ratio (HR)
dNLR
P overall < 0.001
P nonlinearity < 0.001
Hazard Ratio (HR)
SIRI
P overall < 0.001
P nonlinearity = 0.009
Hazard Ratio (HR)
MLR
P overall < 0.001
P nonlinearity = 0.022
Hazard Ratio (HR)

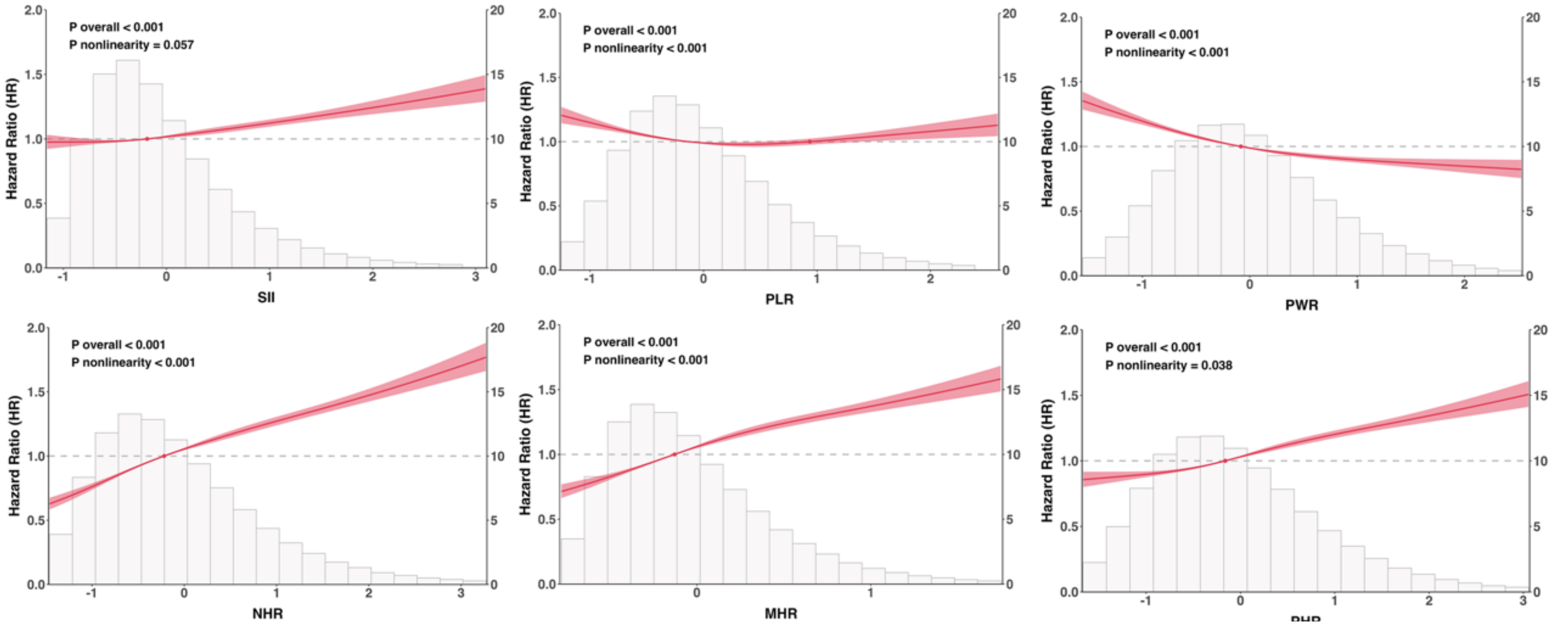

Figure S2. Restricted cubic spline plots depicting the associations between inflammatory markers and the risk of coronary artery disease (CAD). Hazard ratios (HRs) and 95% confidence intervals (shaded areas) were estimated using Cox proportional hazards models with restricted cubic splines. All models were adjusted for covariates. X-axis values represent standardized levels of each marker.

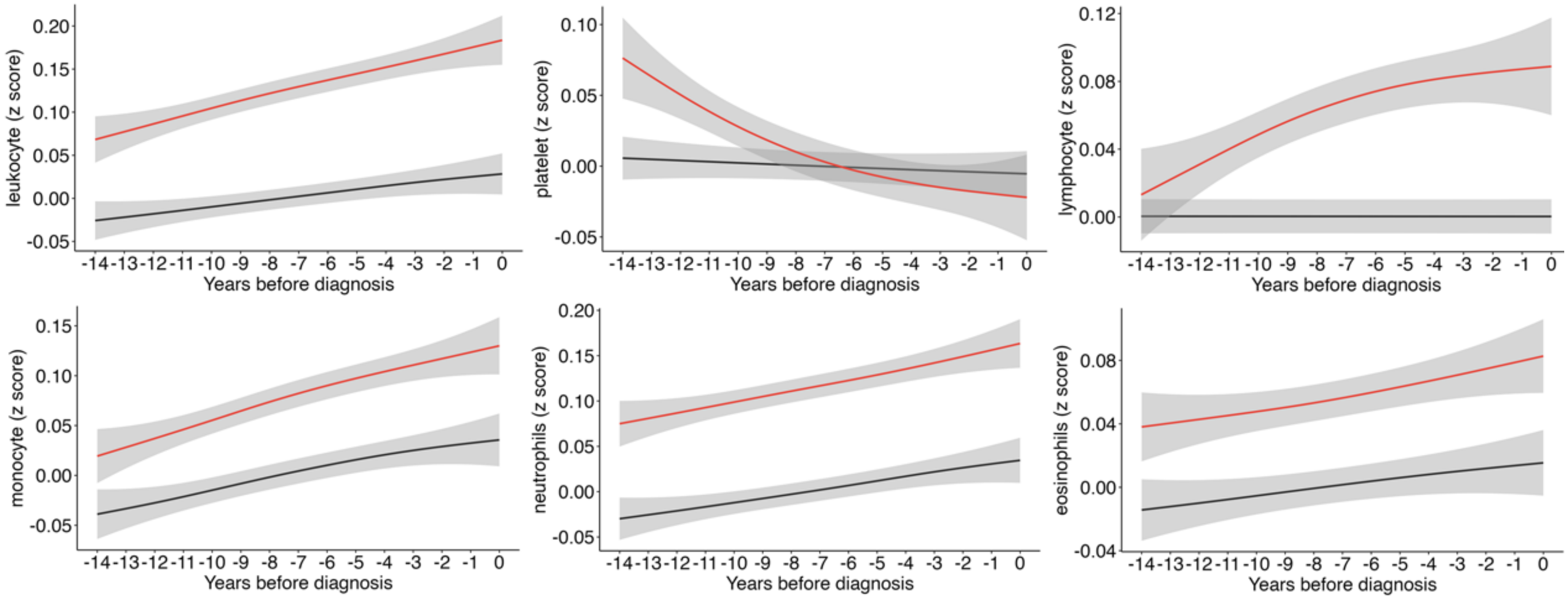
leukocyte (z score)
Years before diagnosis
platelet (z score)
Years before diagnosis
lymphocyte (z score)
Years before diagnosis
monocyte (z score)
Years before diagnosis
neutrophils (z score)
Years before diagnosis
eosinophils (z score)
Years before diagnosis

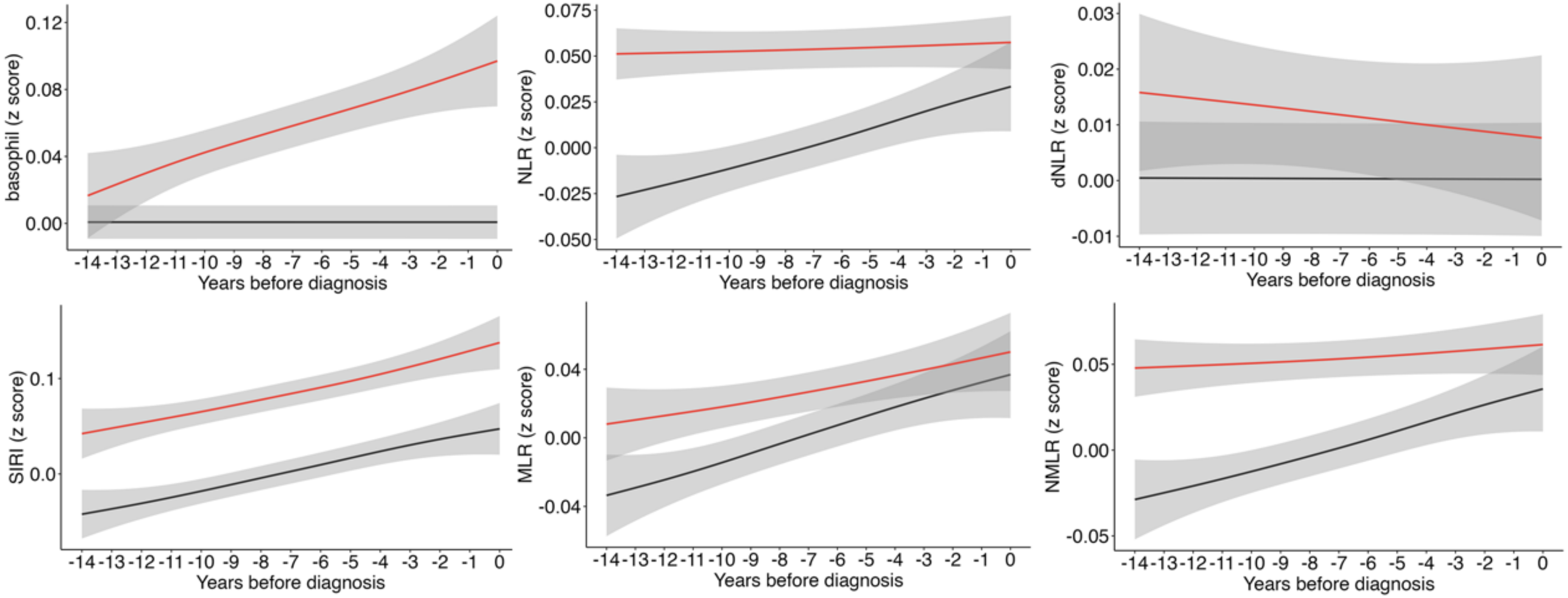
basophil (z score)
0.12
0.08
0.04
0.00
NLR (z score)
0.075
0.050
0.025
0.000
-0.025
-0.050
dNLR (z score)
0.03
0.02
0.01
0.00
-0.01
SIRI (z score)
0.1
0.0
MLR (z score)
0.04
0.00
-0.04
NMLR (z score)
0.05
0.00
-0.05
-14 -13 -12 -11 -10 -9 -8 -7 -6 -5 -4 -3 -2 -1 0
Years before diagnosis

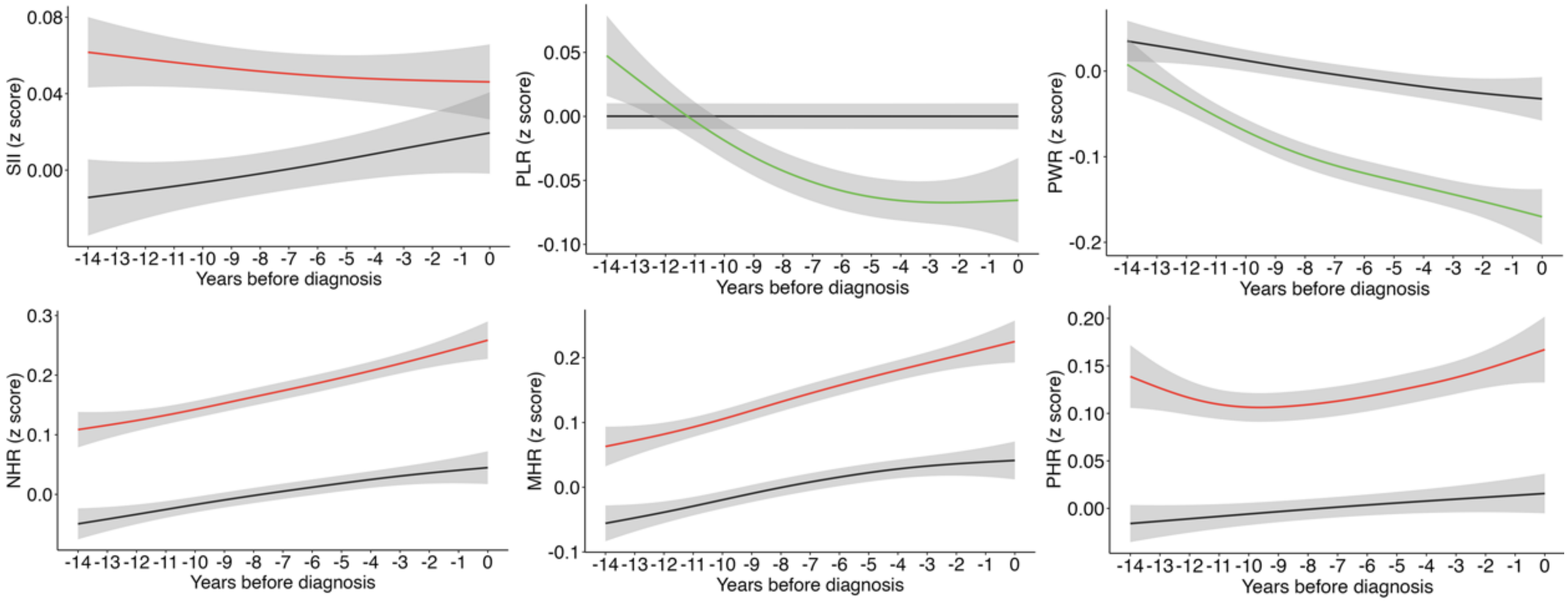

Figure S3. Trends of inflammatory markers in the 14 years prior to coronary artery disease (CAD) diagnosis. The black line represents the control group, while the others represent the CAD group. The x-axis indicates the years before CAD diagnosis, and the y-axis shows the inflammation marker levels standardized by *Z*-score.

Comparison with FRS

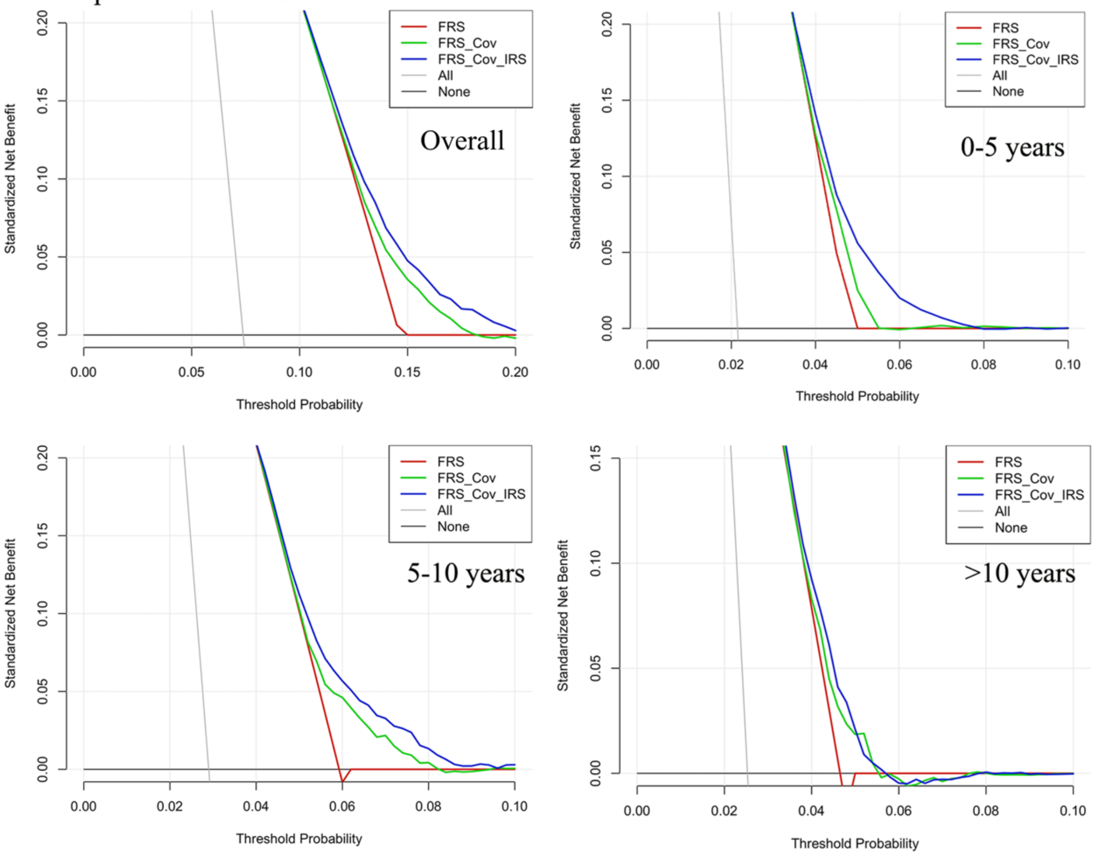

Comparison with SCORE2

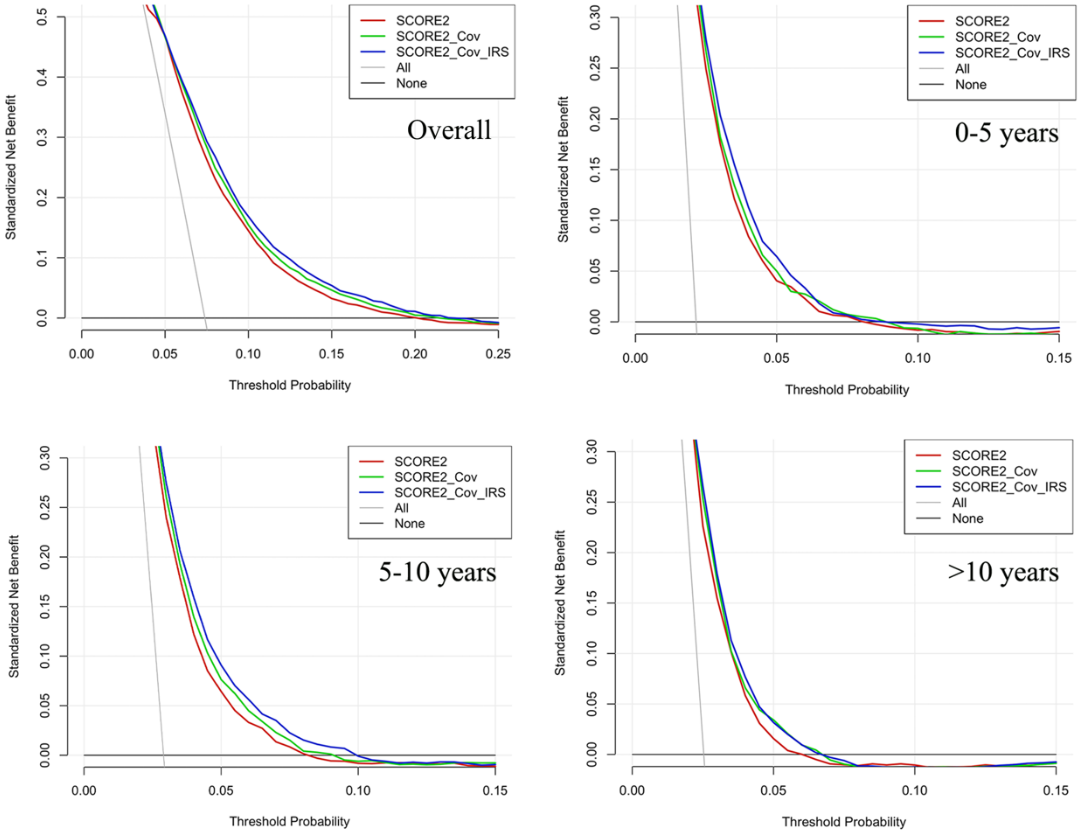

Figure S4. Decision curve analysis (DCA) for CAD risk prediction using FRS and

SCORE2-based models at different time windows (overall, 0-5 years, 5-10 years, and >10 years). The figure showed comparisons based on the Framingham Risk Score (FRS; top row) and SCORE2 (bottom row). Models include: (i) model with only FRS or SCORE2; (ii) model with FRS or SCORE2 as well as other available covariates, which excluded age, sex, and smoking status as they were already included in FRS or SCORE2; (iii) model with FRS or SCORE2, covariates and IRS. The y-axis indicates net benefit; the x-axis shows the threshold probability (i.e., the probability at which a patient would choose to initiate prevention).

Table S1. Outline of JoGH guideline items.

JoGH guideline item	Author's Response
1. Please list all papers published by each co-author in previous 3 years that were based on secondary analysis of a big data repository	1. Hao Zhang, Keying Chen, Tongyu Gao, Yu Yan, Ying Liu, Yuxin Liu, Kexuan Zhu, Jike Qi, Chu Zheng, Ting Wang# and Ping Zeng# (2025). Establishing a robust triangulation framework to explore the relationship between hearing loss and Parkinson's disease. npj Parkinson's Disease, 11(1): 5. https://doi.org/10.1038/s41531-024-00861-5.<br><br>2. Yuxin Liu$, Wenyan Hou$, Tongyu Gao, Yu Yan, Ting Wang, Chu Zheng and Ping Zeng# (2025). Influence and role of polygenic risk score in the development of 32 complex diseases. Journal of Global Health, 15: 04071. https://doi.org/10.7189/jogh.15.04071.<br><br>3. Xin Zhang, Yu Yan, Yuxin Liu, Zixin Wang, Yuchen Jiang, Shuo Zhang, Tongda Xu, Ke Wang, Chu Zheng and Ping Zeng# (2025). Association of biological aging acceleration transitions and burdens with incident cardiovascular disease: longitudinal insights from a national cohort study. BMC Medicine, 23(1): 347. https://doi.org/10.1186/s12916-025-04177-w.<br><br>4. Yuxin Liu, Yu Yan, Yuchen Jiang, Xinyi Wang, Hua Lin, Keying Chen, Shuo Zhang, Fengjun Guan, Pan Zhang, Ting Wang, Ke Wang, Chu Zheng, Yue Xu# and Ping Zeng# (2025). A comprehensive exploration of the impact and contribution of polygenic risk score on age at onset of 30 complex diseases. Public Health, 244: 105754. https://doi.org/10.1016/j.puhe.2025.105754.<br><br>5. Zhou Jiang, Yu Yan, Jike Qi, Yuxin Liu, Yuchen Jiang, Hao Zhang, Hao Chen, Xinying Guan, Pan Zhang, Ting Wang and Ping Zeng# (2025). Roles of genetic predisposition and mediation of biological age acceleration in the association between air pollution and dementia. Journals of Gerontology Series A: Biomedical Sciences and Medical Sciences, 80(6): glaf046. https://doi.org/10.1093/gerona/glaf046.<br><br>6. Shuo Zhang, Ting Wang and Ping Zeng# (2025). Associations of Maternal Smoking During Pregnancy and Genetic Susceptibility with Incident Asthma from a Cohort Study. Prevention Science, 26(3): 343-354. https://doi.org/10.1007/s11121-025-01793-z.<br><br>7. Wenyan Hou$, Yuxin Liu$, Xingjie Hao$, Jike Qi, Yuchen Jiang, Shuiping Huang and Ping Zeng# (2025). Relatively independent and complementary roles of family history and polygenic risk score in age at onset and incident cases of 12 common diseases. Social Science and Medicine, 371: 117942. https://doi.org/10.1016/j.socscimed.2025.117942.<br><br>8. Shuo Zhang, Yuxin Liu, Jike Qi, Yu Yan, Tongyu Gao, Xin Zhang, Dong Sun, Ting Wang and Ping Zeng# (2025). Accelerated aging as a mediator of the association between co-exposure to multiple air pollutants and risk of chronic kidney disease. Ecotoxicology and Environmental Safety, 290: 117582. https://doi.org/10.1016/j.ecoenv.2024.117582.<br><br>9. Wenyan Hou$, Fengjun Guan$, Wenying Chen, Jike Qi, Shuiping Huang# and Ping Zeng# (2024). Breastfeeding, genetic susceptibility, and the risk of asthma and allergic diseases in children and

adolescents: a retrospective national population-based cohort study. BMC Public Health, 24(1): 3056. https://doi.org/10.1186/s12889-024-20501-0.

10. Zhou Jiang[$], Shuo Zhang[$], Tongyu Gao, Keying Chen, Yuxin Liu, Ying Liu, Ting Wang and Ping Zeng[#] (2024). More attention should be paid on time-varying environmental exposures in the UK Biobank. European Journal of Preventive Cardiology, 31(11): e85. https://doi.org/10.1093/eurjpc/zwae160.

11. Shuo Zhang[$], Zhou Jiang[$], Hao Zhang, Yuxin Liu, Jike Qi, Yu Yan, Ting Wang and Ping Zeng[#] (2024). Association of cigarette smoking, smoking cessation with the risk of cardiometabolic multimorbidity in the UK Biobank. BMC Public Health, 24(1): 1910. https://doi.org/10.1186/s12889-024-19457-y.

12. Zhou Jiang[$], Shuo Zhang[$], Tongyu Gao, Keying Chen, Yuxin Liu, Ying Liu, Ting Wang and Ping Zeng[#] (2024). Co-exposure to multiple air pollutants, genetic susceptibility, and risk of acute myocardial infarction onset: A cohort analysis of the UK Biobank participants. European Journal of Preventive Cardiology, 31(6): 698-706. https://doi.org/10.1093/eurjpc/zwad384.

13. Shuo Zhang[$], Hongyan Cao[$], Keying Chen, Tongyu Gao, Huashuo Zhao, Chu Zheng, Ting Wang, Ping Zeng[#] and Ke Wang# (2024). Joint exposure to various ambient air pollutants, genetic susceptibility and incident dementia: a prospective analysis in the UK Biobank cohort. International Journal of Public Health, 69: 1606868. https://doi.org/10.3389/ijph.2024.1606868.

14. Yiyang Zhu, Hao Zhang, Jike Qi, Yuxin Liu, Yu Yan, Ting Wang and Ping Zeng[#] (2024). Evaluating causal association of maternal educational attainment on offspring birthweight via observational study and Mendelian randomization analyses. SSM - Population Health, 25: 101587. https://doi.org/10.1016/j.ssmph.2023.101587.

15. Peilin Yu, Zhou Jiang, Chu Zheng, Ping Zeng, Lihong Huang[#], Yingliang Jin[#] and Ke Wang[#] (2023). Variety ACEs and risk of developing anxiety, depression, or anxiety-depression co-morbidity: the 2006-2022 UK Biobank data. Frontiers in Psychiatry, 14: 1233981. https://doi.org/10.3389/fpsyt.2023.1233981.

16. Zhou Jiang[$], Shuo Zhang[$], Ping Zeng[#] and Ting Wang[#] (2023). Influence of social deprivation on morbidity and all-cause mortality of cardiometabolic multi-morbidity: a cohort analysis of the UK Biobank. BMC Public Health, 23(1): 2177. https://doi.org/10.1186/s12889-023-17008-5.

17. Yuxuan Wu[$], Xingjie Hao[$], Kexuan Zhu, Chu Zheng, Fengjun Guan, Ping Zeng[#] and Ting Wang[#] (2023). Long-term adverse influence of smoking during pregnancy on height and body size of offspring at ten years old in the UK Biobank cohort. SSM - Population Health, 24: 101506. https://doi.org/10.1016/j.ssmph.2023.101506.

18. Zhou Jiang[$], Shuo Zhang[$], Keying Chen, Yuxuan Wu, Ping Zeng[#] and Ting Wang[#] (2023). Long-term influence of air pollutants on morbidity and all-cause mortality of cardiometabolic multi-morbidity: A cohort analysis of the UK Biobank participants. Environmental Research, 237(Part 1): 116873.

	https://doi.org/10.1016/j.envres.2023.116873.
2. Please explain the key elements of your study design and the use of the available datasets that make your study an original scientific contribution	We conducted a large-scale prospective analysis using the UK Biobank cohort to systematically evaluate 18 complete blood count (CBC)-derived inflammatory markers in relation to incident coronary artery disease (CAD). Our study design is original in the following ways: (i) We simultaneously assessed a comprehensive panel of inflammatory markers, including both individual cell counts and derived indices, within a unified analytical framework; (ii) we incorporated mediation analyses to explore the roles of aging and lifestyle in linking inflammation to CAD; (iii) we developed an integrated inflammatory risk score (IRS) and evaluated its added value over established clinical models (FRS, SCORE2) across multiple prediction windows. To our knowledge, no prior study has combined this breadth of biomarkers, methodological triangulation, and predictive assessment using the UK Biobank resource.
3. Please list all publications that addressed similar research questions in the same dataset and indicate where you cited them in your paper	To our knowledge, no prior study using UK Biobank has comprehensively evaluated a wide range of CBC-derived inflammatory markers in relation to incident CAD using both mediation and prediction frameworks.
4. Please explain how you addressed multiple testing through an appropriately rigorous statistical threshold and indicate this in the methods section	We addressed multiple testing by applying a Bonferroni correction for 18 parallel comparisons of inflammatory markers, setting a stringent significance threshold at $P = 2.80 \times 10^{-3}$ (i.e., 0.05/18).
5. Please declare to what extent have AI chatbots been used in developing your paper and to which parts of the paper did they contribute	AI chatbots (e.g., ChatGPT) were used solely for minor language editing and grammar refinement. They were not involved in study design, data analysis, or the generation of scientific content. All final content was written, reviewed, and approved by the authors.

Table S2. Definitions of the exposure, outcome, and covariates from the UK Biobank.

	Variables	Fields	Note
Outcome	CAD	Fields for first occurrence: 131296, 131298, 131300, 131302, 131304, 131306	0, no; 1, yes
Exposure	Leukocyte	30000	
	Platelet	30080	
	Lymphocyte	30120	
	Monocyte	30130	
	Neutrophils	30140	
	Eosinophils	30160	
	Basophils	30170	
	NLR	\	Neutrophils (10^9/L) / lymphocyte (10^9/L)
	dNLR	\	Neutrophils (10^9/L) / [Leukocyte (10^9/L) - lymphocyte (10^9/L)]
	SIRI	\	Neutrophils (10^9/L)×Monocyte (10^9/L) / Lymphocyte (10^9/L)
	MLR	\	Monocyte (10^9/L) / lymphocyte (10^9/L)
	NMLR	\	[Monocyte (10^9/L) + Neutrophils (10^9/L)] / lymphocyte (10^9/L)
	SII	\	Platelet (10^9/L) × Neutrophils (10^9/L) / lymphocyte (10^9/L)
	PLR	\	Platelet (10^9/L) / lymphocyte (10^9/L)
	PWR	\	Platelet (10^9/L) / Leukocyte (10^9/L)
	NHR	\	Neutrophils (10^9/L) / HDL-C (mg/dL)
	MHR	\	Monocyte (10^9/L) / HDL-C (mg/dL)
	PHR	\	Platelet (10^9/L) / HDL-C (mg/dL)
Covariates	Age	21022	
	Sex	31	0, female; 1, male
	BMI	21001	
	Ethnicity	21000	0, white; 1, other
	TDI	189	TDI was derived from the postcode of residence by aggregating data of unemployment, car and home ownership, and household overcrowding.
	Qualification	6138	0, without college; 1, with college

Physical activity	22032	Physical activity was evaluated via the International Physical Activity Questionnaire (IPAQ) short form. Physical activity was divided into three groups: low (<600 min/week), moderate (between 600 and 3000 min/week), and high (>3000 min/week).
Diet score	1289, 1299, 1309, 1319, 1329, 1339, 1349, 1369, 1379, 1389	Healthy diet score was calculated in terms of daily diet factors and ranged from 0 to 5. One point was assigned when met each of the following favorable conditions: 1, vegetable intake ≥four tablespoons/day; 2, fruit intake ≥three pieces/day; 3, fish intake ≥twice/week; 4, unprocessed red meat intake ≤twice/week; 5, and processed meat intake ≤twice/week. Finally, we took the summation of each point as the total healthy diet score.
Drinking history	3731, 20117	0, never drinking; 1, former/current drinking
Smoking history	1249, 20160	0, never smoking; 1, former/current smoking

Note: CAD, coronary artery disease; NLR, neutrophil-to-lymphocyte ratio; dNLR, derived neutrophil-to-lymphocyte ratio; SIRI, systemic inflammation response index; MLR, monocyte-to-lymphocyte ratio; NMLR, neutrophil-to-monocyte-lymphocyte ratio; SII, systemic immune-inflammation index; PLR, platelet-to-lymphocyte ratio; PWR, platelet-to-white blood cell ratio; NHR, neutrophil-to-HDL-C ratio; MHR, monocyte-to-HDL-C ratio; PHR, platelet-to-HDL-C ratio.

Table S3. Sample inclusion before and after exclusion of extreme inflammatory marker values.

Exposure	Before outlier exclusion		After outlier exclusion	
	ALL (*N*)	CAD (*N*)	ALL (*N*)	CAD (*N*)
leukocyte	452219	37920	437411	36366
platelet	452221	37921	437005	36382
lymphocyte	451383	37861	436811	36170
monocyte	451383	37861	439385	36440
neutrophils	451383	37861	436382	36290
eosinophils	451383	37861	442758	36985
basophils	451383	37861	444500	37194
NLR	451376	37861	436103	36215
dNLR	451381	37861	435876	36535
SIRI	451376	37861	436279	36204
MLR	451376	37861	436221	36183
NMLR	451376	37861	436112	36226
SII	451372	37861	436016	36242
PLR	451372	37861	436098	36205
PWR	452213	37920	436766	36605
NHR	395404	33324	382381	31821
MHR	395404	33324	382625	31782
PHR	396134	33375	382742	31921

Note: This table summarizes the total number of participants and CAD cases before and after outlier exclusion.

Table S4. Missingness patterns for clinical covariates in the analysis cohort.

Variable	Missing count (rate, %)
Age	0 (0.00)
Sex	0 (0.00)
BMI	1756 (0.37)
Ethnicity	2174 (0.46)
TDI	582 (0.12)
Qualifications	84078 (17.94)
Physical activity	89701 (19.14)
Diet score	71765 (15.32)
Smoking history	474 (0.10)
Drinking history	477 (0.10)

Note: This table summarizes the missing count and proportion of each clinical covariate included in the main analysis. Multiple imputation was then performed using the Multivariate Imputation by Chained Equations (MICE) framework, generating 5 imputed datasets (m=5), with 20 iterations per chain (maxit=20) to ensure convergence. Imputation methods were selected according to variable type: Continuous variables were imputed using predictive mean matching (PMM) to preserve data distribution; Binary variables were imputed using logistic regression; Nominal categorical variables were imputed using polytomous logistic regression.

Table S5. STROBE Statement—Checklist of items that should be included in reports of cohort studies.

	Item No	Recommendation	Page No
**Title and abstract**	1	(*a*) Indicate the study's design with a commonly used term in the title or the abstract	1
		(*b*) Provide in the abstract an informative and balanced summary of what was done and what was found	2
**Introduction**			
Background/rationale	2	Explain the scientific background and rationale for the investigation being reported	3
Objectives	3	State specific objectives, including any prespecified hypotheses	3-4
**Methods**			
Study design	4	Present key elements of study design early in the paper	Figure1
Setting	5	Describe the setting, locations, and relevant dates, including periods of recruitment, exposure, follow-up, and data collection	6-7
Participants	6	(*a*) Give the eligibility criteria, and the sources and methods of selection of participants. Describe methods of follow-up	6
		(*b*) For matched studies, give matching criteria and number of exposed and unexposed	11
Variables	7	Clearly define all outcomes, exposures, predictors, potential confounders, and effect modifiers. Give diagnostic criteria, if applicable	6-7
Data sources/ measurement	8*	For each variable of interest, give sources of data and details of methods of assessment (measurement). Describe comparability of assessment methods if there is more than one group	6-7
Bias	9	Describe any efforts to address potential sources of bias	7-8
Study size	10	Explain how the study size was arrived at	6
Quantitative variables	11	Explain how quantitative variables were handled in the analyses. If applicable, describe which groupings were chosen and why	6
Statistical methods	12	(*a*) Describe all statistical methods, including those used to control for confounding	7-9
		(*b*) Describe any methods used to examine subgroups and interactions	7-8
		(*c*) Explain how missing data were addressed	10
		(*d*) If applicable, explain how loss to follow-up was addressed	6
		(*e*) Describe any sensitivity analyses	7-8
**Results**			
Participants	13*	(a) Report numbers of individuals at each stage of study—eg numbers potentially eligible, examined for eligibility, confirmed eligible, included in the study, completing follow-up, and analysed	11
		(b) Give reasons for non-participation at each stage	Figure1
		(c) Consider use of a flow diagram	Figure1
Descriptive data	14*	(a) Give characteristics of study participants (eg demographic, clinical, social) and information on exposures and potential confounders	11
		(b) Indicate number of participants with missing data for each variable of interest	Table S4
		(c) Summarise follow-up time (eg, average and total amount)	11
Outcome data	15*	Report numbers of outcome events or summary measures over time	11

Main results	16	(*a*) Give unadjusted estimates and, if applicable, confounder-adjusted estimates and their precision (eg, 95% confidence interval). Make clear which confounders were adjusted for and why they were included	12-13
		(*b*) Report category boundaries when continuous variables were categorized	11
		(*c*) If relevant, consider translating estimates of relative risk into absolute risk for a meaningful time period	12-13
Other analyses	17	Report other analyses done—eg analyses of subgroups and interactions, and sensitivity analyses	14-15
**Discussion**			
Key results	18	Summarise key results with reference to study objectives	20
Limitations	19	Discuss limitations of the study, taking into account sources of potential bias or imprecision. Discuss both direction and magnitude of any potential bias	23
Interpretation	20	Give a cautious overall interpretation of results considering objectives, limitations, multiplicity of analyses, results from similar studies, and other relevant evidence	23
Generalisability	21	Discuss the generalisability (external validity) of the study results	24
**Other information**			
Funding	22	Give the source of funding and the role of the funders for the present study and, if applicable, for the original study on which the present article is based	\

Note: An Explanation and Elaboration article discusses each checklist item and gives methodological background and published examples of transparent reporting. The STROBE checklist is best used in conjunction with this article (freely available on the Web sites of PLoS Medicine at http://www.plosmedicine.org/, Annals of Internal Medicine at http://www.annals.org/, and Epidemiology at http://www.epidem.com/). Information on the STROBE Initiative is available at http://www.strobe-statement.org.

Table S6. Baseline characteristics of the participants analyzed in our study.

Characteristics	All (N=475,134)	Non-cases (N=435,171)	CAD cases (N=39,963)	P
Continuous variables, mean (SD)				
Leukocyte (10^9 cells/L)	6.9 (2.1)	6.8 (2.1)	7.2 (2.3)	5.78×10^{-231}
Platelet (10^9 cells/L)	253.7 (59.8)	254.1 (59.5)	250.2 (62.4)	1.52×10^{-31}
Lymphocyte (10^9 cells/L)	2.0 (1.2)	2.0 (1.1)	2.0 (1.4)	0.99
Monocyte (10^9 cells/L)	0.5 (0.3)	0.5 (0.3)	0.5 (0.2)	0.99
Neutrophils (10^9 cells/L)	4.2 (1.4)	4.2 (1.4)	4.5 (1.5)	3.24×10^{-302}
Eosinophils (10^9 cells/L)	0.2 (0.1)	0.2 (0.1)	0.2 (0.1)	0.99
Basophils (10^9 cells/L)	0.03 (0.1)	0.03 (0.1)	0.03 (0.1)	0.99
NLR	2.3 (1.2)	2.3 (1.2)	2.5 (1.3)	4.20×10^{-181}
dNLR	0.9 (0.1)	0.9 (0.1)	0.9 (0.1)	0.99
SIRI	1.1 (1.1)	1.1 (1.1)	1.3 (0.9)	$<2.20\times10^{-308}$
MLR	0.3 (0.3)	0.3 (0.3)	0.3 (0.2)	0.99
NMLR	2.6 (1.3)	2.6 (1.3)	2.8 (1.4)	1.66×10^{-156}
SII	598.2 (360.6)	595.6 (355.6)	625.9 (410.1)	7.27×10^{-44}
PLR	142.5 (68.5)	142.8 (69.1)	138.8 (60.7)	8.93×10^{-34}
PWR	39 (14.0)	39.2 (14.2)	36.3 (11.1)	$<2.20\times10^{-308}$
NHR	3.1 (1.5)	3.1 (1.4)	3.6 (1.6)	$<2.20\times10^{-308}$
MHR	0.4 (0.3)	0.3 (0.4)	0.4 (0.2)	$<2.20\times10^{-308}$
PHR	185 (64.5)	183.8 (63.8)	198.3 (69.9)	3.75×10^{-287}
Age	56.2 (8.1)	55.9 (8.1)	60.0 (7.0)	$<2.20\times10^{-308}$
BMI	27.3 (4.8)	27.2 (4.7)	28.6 (5)	$<2.20\times10^{-308}$
TDI	-1.3 (3.1)	-1.4 (3.1)	-1.0 (3.2)	2.80×10^{-126}
Follow-up years (years)	13.0 (2.2)	13.5 (0.9)	7.5 (3.9)	\
Categorical variables, N (%)				
Sex				$<2.20\times10^{-308}$
female	264751 (55.7)	249563 (57.3)	15188 (38)	
male	210383 (44.3)	185608 (42.7)	24775 (62)	
Ethnicity				0.67

white	449026 (94.5)	411278 (94.5)	37748 (94.5)	
other	26108 (5.5)	23893 (5.5)	2215 (5.5)	
Qualifications				2.24×10^{-141}
without college degree	292020 (61.5)	265101 (60.9)	26919 (67.4)	
with college degree	183114 (38.5)	170070 (39.1)	13044 (32.6)	
Physical activity				$<2.20\times10^{-308}$
low	89278 (18.8)	80869 (18.6)	8409 (21)	
moderate	193671 (40.8)	177892 (40.9)	15779 (39.5)	
high	192185 (40.4)	176410 (40.5)	15775 (39.5)	
Diet score				$<2.20\times10^{-308}$
0	8707 (1.8)	7755 (1.8)	952 (2.4)	
1	41366 (8.7)	37269 (8.6)	4097 (10.3)	
2	93008 (19.6)	84431 (19.4)	8577 (21.5)	
3	130664 (27.5)	119486 (27.5)	11178 (28)	
4	128852 (27.1)	118786 (27.3)	10066 (25.2)	
5	72537 (15.3)	67444 (15.5)	5093 (12.7)	
Smoking history				1.71×10^{-164}
no	192667 (40.6)	179030 (41.1)	13637 (34.1)	
yes	282467 (59.4)	256141 (58.9)	26326 (65.9)	
Drinking history				3.96×10^{-68}
no	457416 (96.3)	419576 (96.4)	37840 (94.7)	
yes	17718 (3.7)	15595 (3.6)	2123 (5.3)	

Note: Data are represented by mean ± standard deviation (SD) and *N* (%). NLR, Neutrophil-to-lymphocyte ratio; dNLR, Derived neutrophil-to-lymphocyte ratio; SIRI, Systemic inflammation response index; MLR, Monocyte-to-lymphocyte ratio; NMLR, Neutrophil-to-monocyte-lymphocyte ratio; SII, Systemic immune-inflammation index; PLR, Platelet-to-lymphocyte ratio; PWR, Platelet-to-white blood cell ratio; NHR, Neutrophil-to-HDL-C ratio; MHR, Monocyte-to-HDL-C ratio; PHR, Platelet-to-HDL-C ratio.

Table S7. Sensitivity analyses of CAD risk models with inflammatory markers: excluding self-reported outcomes, sex-specific outlier handling and complete-case analysis.

Exposure	Model 1 (Strict outcome definition)	Model 2 (Sex-specific outlier handling)	Model 3 (Complete-case analysis)
leukocyte	1.193 (1.175~1.210), 2.29×10^{-122}	1.204 (1.188~1.220), 1.08×10^{-165}	1.199 (1.177~1.222), 4.69×10^{-79}
platelet	1.031 (1.018~1.045), 5.20×10^{-06}	1.030 (1.018~1.042), 1.35×10^{-06}	1.036 (1.018~1.053), 4.65×10^{-05}
lymphocyte	1.156 (1.129~1.184), 1.93×10^{-33}	1.151 (1.126~1.176), 1.39×10^{-37}	1.142 (1.107~1.177), 1.31×10^{-17}
monocyte	1.156 (1.133~1.179), 5.67×10^{-46}	1.169 (1.148~1.191), 2.08×10^{-64}	1.162 (1.133~1.192), 5.84×10^{-31}
neutrophils	1.142 (1.128~1.157), 2.43×10^{-97}	1.152 (1.139~1.165), 6.14×10^{-135}	1.156 (1.137~1.174), 1.26×10^{-70}
eosinophils	1.065 (1.051~1.080), 2.85×10^{-20}	1.069 (1.056~1.082), 1.09×10^{-26}	1.061 (1.043~1.079), 1.37×10^{-11}
basophil	1.064 (1.047~1.081), 3.29×10^{-14}	1.096 (1.080~1.113), 4.49×10^{-34}	1.100 (1.078~1.122), 1.75×10^{-20}
NLR	1.075 (1.058~1.092), 1.75×10^{-19}	1.083 (1.068~1.099), 2.19×10^{-29}	1.091 (1.070~1.113), 9.62×10^{-18}
dNLR	1.022 (1.008~1.037), 2.21×10^{-03}	1.023 (1.010~1.036), 4.78×10^{-04}	1.032 (1.014~1.050), 5.48×10^{-04}
SIRI	1.174 (1.149~1.200), 1.13×10^{-46}	1.193 (1.170~1.216), 3.97×10^{-72}	1.198 (1.165~1.232), 1.54×10^{-36}
MLR	1.074 (1.037~1.112), 6.29×10^{-05}	1.096 (1.062~1.131), 9.17×10^{-09}	1.103 (1.055~1.153), 1.36×10^{-05}
NMLR	1.075 (1.057~1.093), 5.00×10^{-18}	1.085 (1.070~1.101), 4.20×10^{-28}	1.093 (1.070~1.116), 9.03×10^{-17}
SII	1.079 (1.063~1.096), 4.36×10^{-22}	1.089 (1.074~1.104), 5.24×10^{-33}	1.099 (1.077~1.121), 1.41×10^{-20}
PLR	0.959 (0.943~0.976), 1.88×10^{-06}	0.958 (0.944~0.973), 8.84×10^{-08}	0.964 (0.944~0.986), 1.13×10^{-03}
PWR	0.877 (0.863~0.892), 6.33×10^{-56}	0.869 (0.857~0.882), 4.09×10^{-76}	0.874 (0.857~0.893), 1.35×10^{-37}
NHR	1.226 (1.210~1.243), 4.69×10^{-192}	1.221 (1.207~1.236), 7.97×10^{-231}	1.248 (1.226~1.270), 3.78×10^{-138}
MHR	1.368 (1.332~1.405), 1.47×10^{-118}	1.366 (1.334~1.399), 8.34×10^{-148}	1.386 (1.340~1.434), 1.26×10^{-79}
PHR	1.155 (1.139~1.171), 2.35×10^{-96}	1.144 (1.130~1.158), 2.08×10^{-102}	1.159 (1.139~1.179), 7.80×10^{-62}

Note: Model 1 (Strict outcome definition; self-report excluded): CAD was defined exclusively by ICD-coded diagnoses (I20–I25); cases identified solely by self-report (code 1075) were excluded. Under this stricter definition, 33,004 CAD cases remained. Model 2 (Sex-specific outlier handling): Within males and females separately, outliers were defined and excluded using sex-specific P1/P99 thresholds (thresholds computed within sex strata and then applied), to account for sex-related distributional differences. Model 3 (Complete-case analysis)：a complete-case analysis using participants with no missing covariate data, without any imputation.

Table S8. Sensitivity analyses of CAD risk models with inflammatory markers: medication adjustment, disease exclusions, and removal of early events.

Exposure	Model 1 (Medication-adjusted)	Model 2 (Disease-excluded)	Model 3 (Early-event excluded)
leukocyte	1.185 (1.169~1.201), 5.28×10^{-137}	1.208 (1.189~1.227), 5.94×10^{-121}	1.199 (1.183~1.216), 6.83×10^{-141}
platelet	1.032 (1.020~1.045), 1.34×10^{-07}	1.026 (1.012~1.041), 3.58×10^{-04}	1.030 (1.017~1.043), 5.41×10^{-06}
lymphocyte	1.139 (1.115~1.164), 1.02×10^{-32}	1.158 (1.129~1.189), 1.04×10^{-28}	1.148 (1.122~1.174), 1.71×10^{-32}
monocyte	1.151 (1.130~1.172), 1.93×10^{-52}	1.162 (1.137~1.187), 4.52×10^{-42}	1.167 (1.145~1.190), 2.04×10^{-56}
neutrophils	1.138 (1.126~1.151), 1.66×10^{-111}	1.156 (1.140~1.172), 5.09×10^{-95}	1.149 (1.135~1.163), 6.07×10^{-115}
eosinophils	1.062 (1.049~1.075), 4.05×10^{-22}	1.073 (1.057~1.089), 1.46×10^{-20}	1.063 (1.050~1.077), 1.72×10^{-20}
basophil	1.094 (1.079~1.110), 1.55×10^{-35}	1.092 (1.073~1.112), 1.26×10^{-22}	1.088 (1.072~1.105), 2.17×10^{-28}
NLR	1.075 (1.060~1.091), 6.68×10^{-24}	1.071 (1.052~1.090), 5.15×10^{-14}	1.083 (1.067~1.100), 1.10×10^{-25}
dNLR	1.018 (1.006~1.031), 4.87×10^{-03}	1.016 (1.000~1.031), 4.48×10^{-02}	1.023 (1.009~1.037), 9.96×10^{-04}
SIRI	1.170 (1.147~1.193), 6.66×10^{-54}	1.184 (1.154~1.215), 8.57×10^{-39}	1.191 (1.166~1.217), 6.69×10^{-60}
MLR	1.079 (1.045~1.113), 2.68×10^{-06}	1.060 (1.019~1.103), 3.63×10^{-03}	1.098 (1.062~1.135), 4.63×10^{-08}
NMLR	1.077 (1.061~1.093), 7.70×10^{-23}	1.071 (1.051~1.091), 4.42×10^{-13}	1.085 (1.068~1.102), 1.41×10^{-24}
SII	1.081 (1.066~1.096), 1.49×10^{-27}	1.078 (1.059~1.097), 1.11×10^{-16}	1.084 (1.068~1.100), 2.16×10^{-26}
PLR	0.964 (0.949~0.979), 4.58×10^{-06}	0.954 (0.936~0.972), 8.48×10^{-07}	0.957 (0.941~0.973), 1.79×10^{-07}
PWR	0.884 (0.871~0.897), 4.45×10^{-60}	0.875 (0.860~0.890), 2.34×10^{-50}	0.873 (0.860~0.887), 1.15×10^{-64}
NHR	1.210 (1.195~1.225), 4.29×10^{-201}	1.238 (1.219~1.257), 2.35×10^{-164}	1.226 (1.210~1.242), 7.42×10^{-207}
MHR	1.336 (1.304~1.369), 2.99×10^{-122}	1.402 (1.361~1.445), 1.69×10^{-109}	1.367 (1.333~1.403), 1.22×10^{-127}
PHR	1.138 (1.124~1.152), 7.10×10^{-94}	1.155 (1.138~1.172), 2.03×10^{-79}	1.145 (1.130~1.160), 7.60×10^{-92}

Note: Model 1 (Medication-adjusted): Adds baseline medication indicators (antiplatelet, statins, anticoagulants) to the main covariate set. Antiplatelet (UKB codes): 1140860806 (aspirin 75 mg), 1140864860 (nu-seals aspirin 75 mg e/c), 1140868226 (aspirin), 1141168318 (clopidogrel), 1141167844 (dipyridamole+aspirin), 1140861778 (dipyridamole). Statins: 1140861958 (simvastatin), 1141146234 (atorvastatin), 1140888648 (pravastatin), 1141192410 (rosuvastatin), 1140888594 (fluvastatin). Anticoagulants: 1140888266 (warfarin), 1140910832 (sodium warfarin). Model 2 (Disease-excluded): Excludes participants with baseline history of: Haematological malignancies (ICD-10: C81-C96; e.g., Hodgkin and non-Hodgkin lymphomas, NK/T-cell lymphoma, leukaemias, multiple myeloma);Major chronic liver disease (ICD-10: K70, K72-K76; e.g., alcoholic liver disease, liver failure, chronic hepatitis, cirrhosis, other inflammatory/other liver diseases); Chronic inflammatory

diseases (example ICD-10: rheumatoid arthritis M05-M06; systemic lupus erythematosus M32; Crohn’s disease K50; ulcerative colitis K51).

Model 3 (Early-event removed): Excludes CAD events occurring within 2 years after baseline to reduce reverse causality.

Table S9. Associations between inflammatory markers and CAD risk across quartiles.

Exposure	Q2 vs. Q1 [HR (95%CI), *P*-value]	Q3 vs. Q1 [HR (95%CI), *P*-value]	Q4 vs. Q1 [HR (95%CI), *P*-value]	*P*-trend
leukocyte	1.140 (1.104~1.176), 5.15×10^{-16}	1.239 (1.202~1.277), 4.73×10^{-43}	1.430 (1.387~1.474), 9.54×10^{-120}	9.68×10^{-127}
platelet	0.978 (0.950~1.006), 0.121	0.980 (0.951~1.009), 0.171	1.058 (1.027~1.090), 1.83×10^{-4}	8.73×10^{-4}
lymphocyte	1.009 (0.980~1.040), 0.546	1.040 (1.010~1.072), 9.17×10^{-3}	1.169 (1.136~1.204), 1.14×10^{-25}	3.16×10^{-26}
monocyte	1.080 (1.046~1.114), 2.39×10^{-6}	1.125 (1.090~1.160), 1.45×10^{-13}	1.241 (1.204~1.280), 6.11×10^{-44}	6.27×10^{-46}
neutrophils	1.112 (1.078~1.148), 3.87×10^{-11}	1.209 (1.173~1.247), 2.81×10^{-34}	1.378 (1.337~1.420), 4.77×10^{-98}	8.77×10^{-106}
eosinophils	1.011 (0.972~1.051), 0.591	1.088 (1.060~1.117), 1.86×10^{-10}	1.145 (1.116~1.175), 1.16×10^{-24}	9.52×10^{-27}
basophils	1.103 (1.078~1.128), 7.02×10^{-17}	\	\	\
NLR	1.016 (0.986~1.047), 0.304	1.027 (0.997~1.059), 0.081	1.135 (1.102~1.169), 3.50×10^{-17}	2.43×10^{-17}
dNLR	1.016 (0.987~1.045), 0.281	1.017 (0.988~1.047), 0.245	1.043 (1.013~1.074), 5.30×10^{-3}	7.81×10^{-3}
SIRI	1.070 (1.036~1.105), 4.36×10^{-5}	1.167 (1.131~1.205), 4.73×10^{-22}	1.254 (1.216~1.294), 2.35×10^{-46}	5.71×10^{-54}
MLR	0.994 (0.964~1.026), 0.723	1.020 (0.989~1.052), 0.204	1.048 (1.016~1.081), 3.02×10^{-3}	5.03×10^{-4}
NMLR	1.007 (0.977~1.039), 0.638	1.037 (1.006~1.069), 1.95×10^{-2}	1.131 (1.098~1.165), 3.02×10^{-16}	7.41×10^{-18}
SII	1.010 (0.980~1.041), 0.508	1.044 (1.014~1.076), 4.32×10^{-3}	1.138 (1.106~1.172), 2.71×10^{-18}	1.45×10^{-19}
PLR	0.945 (0.919~0.973), 1.13×10^{-4}	0.921 (0.895~0.949), 3.30×10^{-8}	0.917 (0.890~0.944), 5.98×10^{-9}	7.27×10^{-10}
PWR	0.899 (0.875~0.924), 2.10×10^{-14}	0.821 (0.798~0.845), 4.31×10^{-41}	0.781 (0.757~0.805), 1.21×10^{-56}	3.93×10^{-67}
NHR	1.204 (1.161~1.248), 1.25×10^{-23}	1.365 (1.317~1.414), 2.82×10^{-66}	1.636 (1.579~1.695), 4.30×10^{-165}	3.72×10^{-182}
MHR	1.171 (1.128~1.214), 4.70×10^{-17}	1.284 (1.239~1.332), 1.03×10^{-41}	1.528 (1.474~1.585), 2.63×10^{-115}	2.36×10^{-125}
PHR	1.080 (1.044~1.118), 8.61×10^{-6}	1.196 (1.157~1.237), 4.79×10^{-26}	1.361 (1.317~1.407), 6.18×10^{-75}	7.38×10^{-86}

Note: HR, hazard ratio; CI, confidence interval. HR and 95%CI were estimated using Cox models for the second, third and fourth quartiles (Q2-Q4), with the first quartile (Q1) as the reference. *P* for trend was calculated by treating quartiles as an ordinal variable. For basophil count, due to a high proportion of zero values, participants were categorized into two groups: undetectable (Q1, value=0) and detectable (Q2, value>0).

Table S10. Relation between inflammatory markers and CAD risk in the baseline population stratified by sex.

Exposure	Female [HR (95%CI), *P*-value]	Male [HR (95%CI), *P*-value]	*Z* test
leukocyte	1.231 (1.204~1.258), 9.97×10^{-79}	1.196 (1.176~1.217), 4.37×10^{-96}	4.23×10^{-2}
platelet	1.030 (1.011~1.050), 1.73×10^{-3}	1.031 (1.015~1.047), 1.10×10^{-4}	0.94
lymphocyte	1.151 (1.113~1.191), 5.36×10^{-16}	1.142 (1.111~1.174), 5.70×10^{-21}	0.72
monocyte	1.200 (1.163~1.238), 1.95×10^{-30}	1.163 (1.137~1.189), 1.55×10^{-40}	0.11
neutrophils	1.179 (1.157~1.201), 5.67×10^{-68}	1.148 (1.132~1.165), 1.95×10^{-80}	2.66×10^{-2}
eosinophils	1.074 (1.052~1.097), 2.08×10^{-11}	1.067 (1.051~1.083), 3.91×10^{-17}	0.62
basophil	1.094 (1.070~1.119), 2.85×10^{-15}	1.099 (1.079~1.120), 4.21×10^{-24}	0.76
NLR	1.120 (1.093~1.148), 6.18×10^{-20}	1.080 (1.061~1.098), 6.27×10^{-18}	1.73×10^{-2}
dNLR	1.029 (1.007~1.051), 1.03×10^{-2}	1.020 (1.004~1.036), 1.31×10^{-2}	0.52
SIRI	1.277 (1.232~1.323), 3.44×10^{-41}	1.177 (1.149~1.206), 4.84×10^{-41}	2.07×10^{-4}
MLR	1.153 (1.089~1.221), 1.07×10^{-6}	1.089 (1.049~1.131), 1.07×10^{-5}	0.10
NMLR	1.125 (1.097~1.154), 1.55×10^{-19}	1.082 (1.062~1.101), 2.89×10^{-17}	1.40×10^{-2}
SII	1.117 (1.092~1.143), 4.56×10^{-21}	1.081 (1.062~1.101), 4.87×10^{-18}	2.73×10^{-2}
PLR	0.967 (0.943~0.991), 8.69×10^{-3}	0.957 (0.938~0.976), 1.52×10^{-5}	0.52
PWR	0.864 (0.844~0.884), 7.69×10^{-36}	0.868 (0.852~0.885), 4.37×10^{-46}	0.76
NHR	1.302 (1.275~1.330), 2.71×10^{-131}	1.206 (1.188~1.224), 1.46×10^{-132}	6.42×10^{-9}
MHR	1.564 (1.496~1.635), 1.31×10^{-86}	1.320 (1.283~1.359), 4.32×10^{-81}	3.36×10^{-10}
PHR	1.182 (1.158~1.207), 1.01×10^{-56}	1.131 (1.114~1.149), 6.89×10^{-56}	8.28×10^{-4}

Note: This table presents the hazard ratio (HR) estimates, 95% confidence intervals (CI), and P-values for the association between inflammatory markers and coronary artery disease (CAD) after stratified by sex. Z test was used to compare the difference between HRs, calculated as $(\mathrm{logHR}_{\mathrm{male}} - \mathrm{logHR}_{\mathrm{female}})\ /\ \mathrm{sqrt}\ (se^2_{\mathrm{male}} + se^2_{\mathrm{female}})$, where se was the standard error. A P-value $< 2.8\times10^{-3}$ was considered statistically significant.

Table S11. Relation between inflammatory markers and CAD risk in the baseline population stratified by age.

Exposure	Age≤55 years [HR (95%CI), *P*-value]	Age>55 years [HR (95%CI), *P*-value]	*Z* test
leukocyte	1.276 (1.243~1.310), 2.14×10^{-74}	1.186 (1.168~1.204), 1.49×10^{-103}	2.27×10^{-6}
platelet	1.079 (1.054~1.105), 3.82×10^{-10}	1.014 (1.000~1.028), 0.055	8.58×10^{-6}
lymphocyte	1.306 (1.251~1.364), 7.94×10^{-34}	1.098 (1.071~1.126), 1.16×10^{-13}	1.01×10^{-11}
monocyte	1.187 (1.143~1.231), 1.41×10^{-19}	1.172 (1.148~1.197), 5.58×10^{-51}	0.56
neutrophils	1.208 (1.182~1.235), 3.38×10^{-63}	1.142 (1.127~1.157), 4.80×10^{-87}	1.65×10^{-5}
eosinophils	1.089 (1.063~1.116), 7.14×10^{-12}	1.063 (1.048~1.078), 1.99×10^{-17}	0.09
basophil	1.120 (1.089~1.152), 1.17×10^{-15}	1.088 (1.070~1.106), 8.54×10^{-24}	0.08
NLR	1.094 (1.062~1.127), 3.05×10^{-9}	1.092 (1.074~1.109), 1.83×10^{-26}	0.92
dNLR	1.062 (1.035~1.090), 4.63×10^{-6}	1.009 (0.995~1.024), 0.21	7.01×10^{-4}
SIRI	1.218 (1.168~1.269), 1.20×10^{-20}	1.203 (1.176~1.230), 1.29×10^{-57}	0.61
MLR	1.009 (0.942~1.081), 0.788	1.138 (1.098~1.180), 1.21×10^{-12}	2.40×10^{-3}
NMLR	1.097 (1.064~1.132), 4.26×10^{-9}	1.094 (1.076~1.113), 1.35×10^{-25}	0.88
SII	1.111 (1.079~1.143), 8.09×10^{-13}	1.087 (1.070~1.105), 2.20×10^{-24}	0.19
PLR	0.927 (0.897~0.957), 4.05×10^{-6}	0.971 (0.953~0.988), 9.77×10^{-4}	1.42×10^{-2}
PWR	0.849 (0.825~0.874), 3.03×10^{-28}	0.872 (0.857~0.887), 6.40×10^{-55}	0.12
NHR	1.292 (1.261~1.324), 2.36×10^{-96}	1.218 (1.201~1.236), 1.54×10^{-161}	4.39×10^{-5}
MHR	1.417 (1.349~1.488), 1.49×10^{-44}	1.376 (1.338~1.414), 4.49×10^{-113}	0.31
PHR	1.205 (1.176~1.235), 6.99×10^{-51}	1.131 (1.115~1.147), 2.04×10^{-63}	1.11×10^{-5}

Note: This table presents the hazard ratio (HR) estimates, 95% confidence intervals (CI), and P-values for the association between inflammatory markers and coronary artery disease (CAD) after stratified by age at 55 years. *Z* test was used to compare the difference between HRs, calculated as $(\mathrm{logHR}_{\mathrm{age}\le55} - \mathrm{logHR}_{\mathrm{age}>55})$ / sqrt $(se^2_{\mathrm{age}\le55} + se^2_{\mathrm{age}>55})$, where se was the standard error. A *P*-value < 2.8×10^{-3} was considered statistically significant.

Table S12. Time-stratified Cox regression and proportional hazards (PH) assumption testing for inflammatory markers and CAD risk.

Exposure	PH (*P*)	0-5 years [HR (95%CI), *P*-value]	5-10 years [HR (95%CI), *P*-value]	>10 years [HR (95%CI), *P*-value]	*Z* test (0-5 vs. >10 years)
leukocyte	5.53×10^{-07}	1.245 (1.216~1.276), 9.14×10^{-72}	1.209 (1.184~1.236), 8.96×10^{-68}	1.156 (1.129~1.185), 4.65×10^{-32}	2.07×10^{-05}
platelet	0.19	1.034 (1.012~1.057), 2.45×10^{-03}	1.030 (1.010~1.050), 3.06×10^{-03}	1.036 (1.014~1.058), 1.41×10^{-03}	0.90
lymphocyte	0.38	1.171 (1.126~1.217), 1.59×10^{-15}	1.173 (1.134~1.214), 9.73×10^{-20}	1.097 (1.055~1.140), 3.67×10^{-06}	1.97×10^{-02}
monocyte	1.95×10^{-05}	1.191 (1.153~1.231), 5.84×10^{-26}	1.193 (1.159~1.229), 6.99×10^{-33}	1.121 (1.085~1.158), 9.26×10^{-12}	1.01×10^{-02}
neutrophils	2.84×10^{-06}	1.179 (1.155~1.203), 1.28×10^{-56}	1.159 (1.139~1.181), 1.52×10^{-57}	1.120 (1.098~1.144), 1.35×10^{-27}	5.00×10^{-04}
eosinophils	1.69×10^{-02}	1.081 (1.058~1.105), 3.46×10^{-12}	1.060 (1.039~1.081), 6.87×10^{-09}	1.066 (1.042~1.089), 1.51×10^{-08}	0.38
basophil	0.20	1.109 (1.081~1.139), 8.16×10^{-15}	1.079 (1.053~1.105), 4.07×10^{-10}	1.090 (1.062~1.117), 2.68×10^{-11}	0.35
NLR	3.48×10^{-02}	1.089 (1.062~1.117), 5.47×10^{-11}	1.081 (1.056~1.106), 2.38×10^{-11}	1.089 (1.061~1.117), 6.31×10^{-11}	0.99
dNLR	0.51	1.027 (1.003~1.051), 2.49×10^{-02}	1.021 (1.001~1.043), 4.34×10^{-02}	1.014 (0.991~1.037), 2.39×10^{-01}	0.44
SIRI	2.55×10^{-04}	1.205 (1.163~1.249), 8.94×10^{-25}	1.210 (1.172~1.249), 7.75×10^{-32}	1.165 (1.124~1.208), 1.20×10^{-16}	0.19
MLR	2.45×10^{-02}	1.085 (1.025~1.149), 4.95×10^{-03}	1.104 (1.050~1.162), 1.33×10^{-04}	1.102 (1.041~1.166), 8.80×10^{-04}	0.71
NMLR	2.65×10^{-02}	1.090 (1.062~1.120), 2.18×10^{-10}	1.085 (1.060~1.112), 1.55×10^{-11}	1.090 (1.061~1.119), 2.93×10^{-10}	0.99
SII	0.07	1.096 (1.069~1.124), 1.16×10^{-12}	1.083 (1.059~1.108), 5.05×10^{-12}	1.091 (1.064~1.119), 1.35×10^{-11}	0.80
PLR	0.17	0.948 (0.921~0.975), 2.31×10^{-04}	0.952 (0.928~0.976), 1.24×10^{-04}	0.983 (0.956~1.011), 2.37×10^{-01}	0.08
PWR	3.27×10^{-07}	0.841 (0.819~0.865), 1.10×10^{-34}	0.861 (0.840~0.882), 4.98×10^{-34}	0.907 (0.883~0.931), 3.03×10^{-13}	9.91×10^{-05}
NHR	2.42×10^{-08}	1.265 (1.237~1.293), 2.53×10^{-98}	1.233 (1.209~1.258), 6.80×10^{-96}	1.195 (1.169~1.222), 2.92×10^{-55}	3.69×10^{-04}
MHR	4.79×10^{-08}	1.438 (1.378~1.501), 5.44×10^{-62}	1.402 (1.349~1.457), 1.35×10^{-65}	1.289 (1.234~1.347), 9.07×10^{-30}	4.61×10^{-04}
PHR	1.09×10^{-03}	1.178 (1.152~1.205), 5.18×10^{-47}	1.147 (1.124~1.170), 4.19×10^{-41}	1.136 (1.110~1.161), 8.55×10^{-29}	2.52×10^{-02}

Note: This table presents hazard ratio (HR) estimates, 95% confidence intervals (CI), and *P*-values for the associations between 18 inflammatory markers and incident coronary artery disease (CAD), stratified by time to CAD onset: 0-5 years, 5-10 years, and >10 years. Also included are the global *P*-values from Schoenfeld residual tests for proportional hazards (PH) assumptions. A PH *P*-value < 0.05 suggests potential time-varying effects over follow-up, in which case stratified Cox models were subsequently applied to capture period-specific hazard estimates. *Z* test was used to compare the difference between HRs, calculated as $(\mathrm{logHR}_{0-5\ \mathrm{years}} - \mathrm{logHR}_{>10\ \mathrm{years}})\ /\ \mathrm{sqrt}\ (se^2_{0-5\ \mathrm{years}} + se^2_{>10\ \mathrm{years}})$, where *se* was the standard error. A *P*-value $<2.8\times10^{-3}$ was considered statistically significant.

Table S13. Key inflammation variables and their estimated coefficient in the LASSO-derived inflammatory score.

Inflammatory markers	platelet	lymphocyte	eosinophils	basophil	NLR	dNLR	NMLR	PWR	NHR	PHR
Coefficient (β)	-0.0204	0.0715	-0.0329	0.0158	0.0491	-0.0790	0.0147	-0.1028	0.0964	0.1382

Note: NLR, neutrophil-to-lymphocyte ratio; dNLR, derived neutrophil-to-lymphocyte ratio; NMLR, neutrophil-to-monocyte-lymphocyte ratio; PWR, platelet-to-white blood cell ratio; NHR, neutrophil-to-HDL-C ratio; PHR, platelet-to-HDL-C ratio.

Table S14. Net Reclassification Improvement (NRI) and number of reclassified individuals across models and time windows at the 7.5% risk threshold.

Target	Model comparison	NRI (95CI %)	Event (*N*)	Event (Up)	Event (Down)	Non-Event (*N*)	Non-Event (Up)	Non-Event (Down)
Overall	FRS vs FRS+Cov	0.7% (0.2~1.1%)	5849	178	0	73324	1743	0
Overall	SCORE2 vs SCORE2+Cov	2.1% (1.2~3.2%)	5849	569	323	73324	4911	3384
Overall	FRS vs FRS+Cov+IRS	0.6% (0.1~1.2%)	5849	243	0	73324	2575	0
Overall	SCORE2 vs SCORE2+Cov+IRS	3.1% (2.0~4.2%)	5849	707	373	73324	6077	4140
Overall	FRS+Cov vs FRS+Cov+IRS	-0.0% (-0.5~0.4%)	5849	117	52	73324	1374	542
Overall	SCORE2+Cov vs SCORE2+Cov+IRS	0.9% (0.1~1.7%)	5849	360	272	73324	3235	2825
0-5years	FRS vs FRS+Cov	0.5% (0.2~1.0%)	1700	12	0	77473	139	0
0-5years	SCORE2 vs SCORE2+Cov	0.6% (-0.2~1.4%)	1700	30	17	77473	356	235
0-5years	FRS vs FRS+Cov+IRS	1.6% (1.0~2.3%)	1700	35	0	77473	350	0
0-5years	SCORE2 vs SCORE2+Cov+IRS	0.9% (-0.1~2.0%)	1700	50	29	77473	573	342
0-5years	FRS+Cov vs FRS+Cov+IRS	1.1% (0.4~1.8%)	1700	29	6	77473	281	70
0-5years	SCORE2+Cov vs SCORE2+Cov+IRS	0.3% (-0.8~1.3%)	1700	36	28	77473	406	296
5-10years	FRS vs FRS+Cov	5.0% (3.8~6.1%)	2246	168	0	75227	1846	0
5-10years	SCORE2 vs SCORE2+Cov	1.3% (0.5~2.3%)	2246	68	30	75227	752	471
5-10years	FRS vs FRS+Cov+IRS	8.5% (7.3~9.8%)	2246	273	0	75227	2751	0
5-10years	SCORE2 vs SCORE2+Cov+IRS	2.7% (1.6~3.6%)	2246	108	31	75227	1102	545
5-10years	FRS+Cov vs FRS+Cov+IRS	3.5% (2.3~4.5%)	2246	136	31	75227	1417	512
5-10years	SCORE2+Cov vs SCORE2+Cov+IRS	1.4% (0.5~2.2%)	2246	72	33	75227	696	420
>10years	FRS vs FRS+Cov	0.4% (0.1~0.8%)	1903	12	0	73324	172	0
>10years	SCORE2 vs SCORE2+Cov	0.6% (-0.0~1.3%)	1903	31	16	73324	379	210
>10years	FRS vs FRS+Cov+IRS	0.4% (0.1~0.8%)	1903	14	0	73324	233	0
>10years	SCORE2 vs SCORE2+Cov+IRS	1.1% (0.3~1.9%)	1903	42	15	73324	465	230
>10years	FRS+Cov vs FRS+Cov+IRS	0.0% (-0.3~0.4%)	1903	6	4	73324	108	47
>10years	SCORE2+Cov vs SCORE2+Cov+IRS	0.5% (-0.0~1.1%)	1903	26	14	73324	244	178

Note: This table presents the Net Reclassification Improvement (NRI) values with 95% confidence intervals and the number of reclassified individuals at a clinical risk threshold of 7.5%. Reclassification is shown separately for cases (Event) and non-cases (NonEvent), including the number of individuals reclassified to a higher risk category (Up) or a lower risk category (Down). Analyses were stratified by time to CAD onset and conducted for different model comparisons.

References

1. Klemera, P. and S. Doubal, *A new approach to the concept and computation of biological age.* Mechanisms of Ageing and Development, 2006. **127**(3): p. 240-248.
2. Li, X., et al., *Accelerated aging mediates the associations of unhealthy lifestyles with cardiovascular disease, cancer, and mortality.* J Am Geriatr Soc, 2024. **72**(1): p. 181-193.
